# Infection-Associated Nuclear Degeneration in the Rice Blast Fungus *Magnaporthe oryzae* Requires Non-Selective Macro-Autophagy

**DOI:** 10.1371/journal.pone.0033270

**Published:** 2012-03-20

**Authors:** Min He, Michael J. Kershaw, Darren M. Soanes, Yuxian Xia, Nicholas J. Talbot

**Affiliations:** 1 School of Biosciences, University of Exeter, Exeter, Devon, United Kingdom; 2 Genetic Engineering Research Center, College of Bioengineering, Chongqing University, Chongqing, People's Republic of China; Seoul National University, Republic of Korea

## Abstract

**Background:**

The rice blast fungus *Magnaporthe oryzae* elaborates a specialized infection structure called an appressorium to breach the rice leaf surface and gain access to plant tissue. Appressorium development is controlled by cell cycle progression, and a single round of nuclear division occurs prior to appressorium formation. Mitosis is always followed by programmed cell death of the spore from which the appressorium develops. Nuclear degeneration in the spore is known to be essential for plant infection, but the precise mechanism by which it occurs is not known.

**Methodology/Principal Findings:**

In yeast, nuclear breakdown requires a specific form of autophagy, known as piecemeal microautophagy of the nucleus (PMN), and we therefore investigated whether this process occurs in the rice blast fungus. Here, we report that *M. oryzae* possesses two conserved components of a putative PMN pathway, MoVac8 and MoTsc13, but that both are dispensable for nuclear breakdown during plant infection. *MoVAC8* encodes a vacuolar membrane protein and *MoTSC13* a peri-nuclear and peripheral ER protein.

**Conclusions/Significance:**

We show that *MoVAC8* is necessary for caffeine resistance, but dispensable for pathogenicity of *M. oryzae*, while *MoTSC13* is involved in cell wall stress responses and is an important virulence determinant. By functional analysis of *ΔMoatg1* and *ΔMoatg4* mutants, we demonstrate that infection-associated nuclear degeneration in *M. oryzae* instead occurs by non-selective macroautophagy, which is necessary for rice blast disease.

## Introduction

Rice blast disease is a widespread constraint to rice production and therefore poses a persistent threat to global food security [1]. Rice blast infections, caused by the ascomycete fungus *Magnaporthe oryzae*, are initiated by attachment of a three-celled spore, or conidium, to the rice leaf cuticle. The conidium sticks tightly to the leaf surface by means of an adhesive released from the spore tip during hydration [2]. Once attached, the conidium quickly germinates and forms a single polarized germ tube. Within 4 hours, the germ tube ceases apical extension and terminal hooking of the hypha starts, which represents the initiation of cellular differentiation to form a specialised dome-shaped cell, the appressorium, that is necessary for successful plant infection [3]. A narrow penetration hypha is formed at the base of the appressorium and enters the underlying epidermis, rupturing the cell wall and invaginating the plant plasma membrane [1].

Development of the *M. oryzae* appressorium requires external cues including a hard, hydrophobic surface and the absence of exogenous nutrients [4]. Multiple cellular signal transduction cascades, such as the cyclic AMP and Pmk1 MAPK signaling pathways, are initiated in response to these external triggers and bring about the terminal differentiation of the germ tube apex into an appressorium [3], [5]. The appressorium of *M. oryzae* ruptures the plant cuticle by application of mechanical force through accumulation of very high concentrations of glycerol, which draws water into the appressorium to create enormous hydrostatic turgor [6]. Autophagic re-cycling of the contents of the conidium is necessary for formation of a functional appressorium [7]. Consistent with this, lipid and glycogen mobilization, under control of the MAPK and cAMP response pathways, have been shown to occur during appressorium development and may provide precursors for glycerol synthesis [8], [9].

It is now clear that appressorium development by *M. oryzae* is genetically controlled by cell cycle progression and that entry of a nucleus in the germinating conidial cell into S-phase is the key step in initiating infection structure development [7], [10]. During germination and appressorium development, one nucleus in the conidium undergoes mitosis in the germ tube, after which one daughter nucleus moves into the incipient appressorium and the other returns to the conidium and degenerates [7]. Completion of mitosis leads to collapse and death of the conidium and is necessary for appressorium maturation and plant infection [11]. Systematic deletion of genes encoding each component of the macroautophagy machinery renders *M. oryzae* non-pathogenic, providing evidence that autophagy is essential for plant infection [7], [11]–[14].

Despite evidence to show the importance of autophagy in programmed cell death of the conidium and subsequent appressorium maturation, the molecular machinery responsible for nuclear degeneration in the conidium of *M. oryzae* remains unknown. Moreover, the factors regulating nuclear degeneration and the destiny of degraded nuclei in the conidium have yet to be characterised. In *S. cerevisiae*, it has been shown that piecemeal microautophagy of the nucleus (PMN) is a separate process that is necessary for recycling of non-essential portions of the nucleus and is induced by starvation or exposure to rapamycin, an inhibitor of the TOR signalling pathway [15], [16], [17], [18], [19], [20], [21]. PMN occurs constitutively at nucleus-vacuole (NV) junctions, formed through a specific binding interaction of Vac8p on the vacuole membrane and Nvj1p in the outer nuclear envelope [15], [16]. During PMN, small teardrop-shaped portions of the nucleus are extruded along NV junctions into invaginations of the vacuolar membrane, which results in formation of tethered blebs that finally release vesicles containing non-essential nuclear material into the vacuole lumen for degradation by resident hydrolases [16], [21]. Lipid metabolic proteins Osh1p and Tsc13p have been shown to be recruited and enriched at NV junctions by physical association with Nvj1p and may function in non-vesicular lipid trafficking and biogenesis of a distinctive lipid environment at NV junctions [19], [22]. In addition, a spectrum of core autophagy machinery genes is required for the terminal vacuolar enclosure of the invaginated blebs and efficient production of intravacuolar PMN vesicles [20].

In this study, we set out to determine whether there is an identifiable PMN pathway in *M. oryzae* and to ask whether this process drives nuclear degeneration in the conidium during rice blast infection. Here, we report that *MoVAC8* encodes a vacuolar membrane protein, which plays a role in the caffeine response, and that *MoTSC13* is necessary for maintaining conidial morphology and for penetration peg development during plant infection. Importantly, we demonstrate that nuclear degeneration in the conidium occurs even in the absence of *MoVAC8* and *MoTSC13* and that there is no evidence for a discernable PMN pathway in *M. oryzae*. Instead *M. oryzae* degrades nuclei using a macroautophagic mechanism, which is a necessary pre-requisite for plant infection.

## Results and Discussion

### Nuclear degeneration occurs during appressorium development in *M. oryzae*

**Figure 1:**
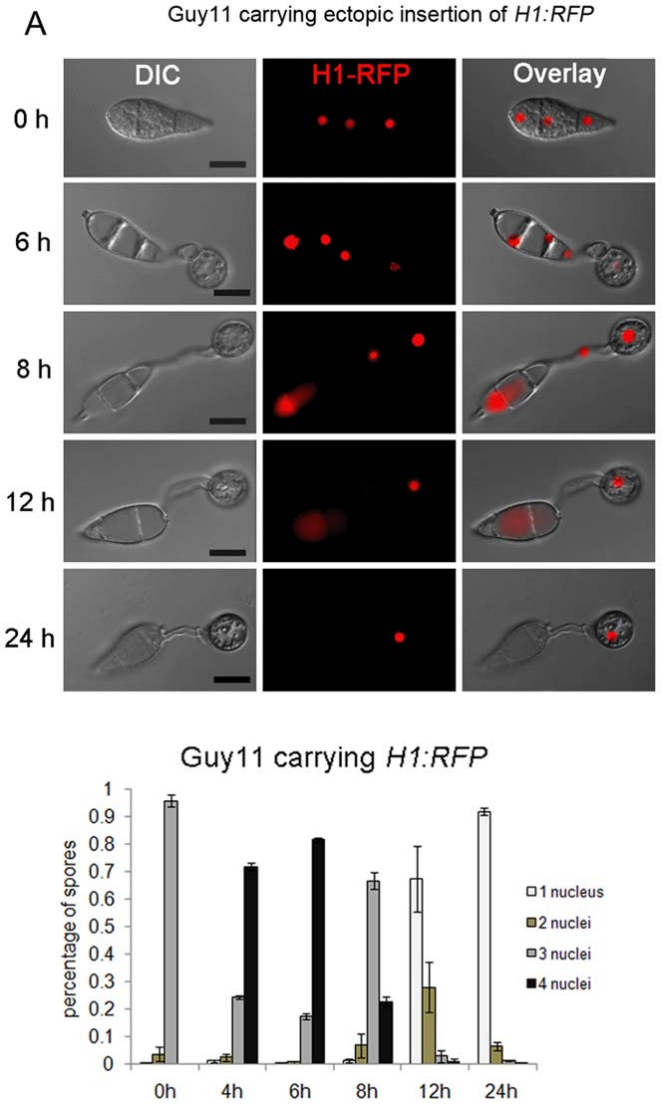
Nuclear degeneration occurs during appressorium development in *M. oryzae*. (**A**). Upper panel: time course live cell imaging experiment showing nuclear division and nuclear degeneration during appressorium development in *M. oryzae*. Guy11 conidia expressing *H1:RFP* were examined by epifluorescence microscopy at indicated time points during appressorium development. Lower panel: bar charts showing the percentage of spore germlings in Guy11 containing between 0 and 4 nuclei (mean ± SD, n>100, triple replications) during a timecourse of appressorium development. Scale bar = 10 µm.

To investigate nuclear behaviour during appressorium development, we performed live-cell imaging and quantitative analysis of nuclear number in a *M. oryzae* strain expressing a histone H1-enhanced red fluorescent (*H1:RFP*) protein fusion [10]. Mitosis occurred in the germ tube emerging from the apical cell of the conidium between 4–6 hour post inoculation (hpi) and the daughter nucleus moved into the incipient appressorium, while the mother nucleus returned to the conidium, as shown in Figure 1. After the completion of mitosis and formation of the appressorium, nuclear degeneration occurred in the conidium, during which the nucleus in the basal cell of the conidium collapsed first, followed by the two nuclei occupying the middle cell and apical cell, respectively, as shown in Figure 1. Nuclear degeneration occurred without overt nuclear fragmentation and red fluorescence associated with nuclear material could be observed both in the cytoplasm and in vacuoles within conidia. After 24 h, nuclear degeneration always resulted in a single nucleus, which was present in the mature appressorium (Figure 1).

### Two components of the piecemeal autophagy of the nucleus pathway are present in the *M. oryzae* genome

**Figure 2:**
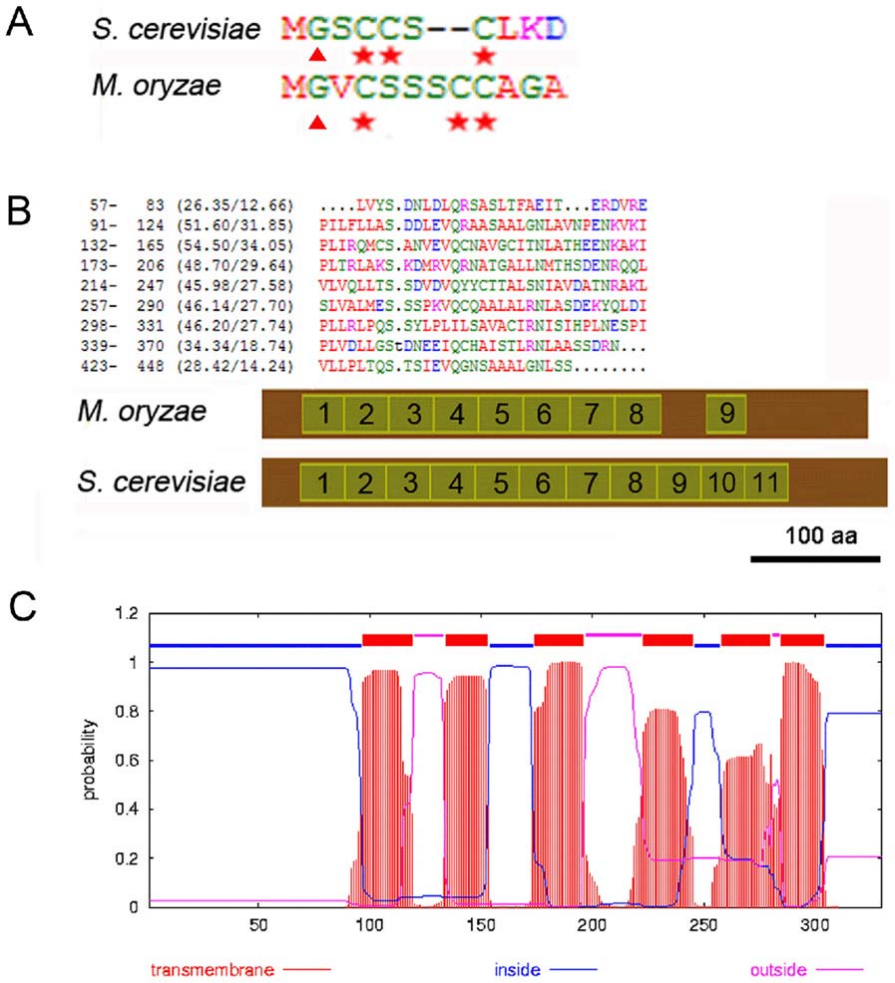
Bioinformatic identification of components of the *S. cerevisiae* PMN pathway in *M. oryzae*. (**A**) Alignment of acylation modification amino acids in the N-terminus of Vac8p from *M. oryzae* and *S. cerevisiae*. Triangles indicate putative myristoylation modification site (Gly) and stars indicate palmitoylation modification sites (Cys) that have been shown to be important for localisation and function of Vac8p in *S. cerevisiae*. Similar acylation modification sites are present in MoVac8p. (**B**) ARM repeat organization in MoVac8p is distinct from that of *S. cerevisiae* Vac8p. Upper panel: alignment of 9 ARM repeats from *M. oryzae* Vac8p. A dot indicates a space. Lower panel: Comparison of distribution of the ARM repeats in Vac8p between *M. oryzae* and *S. cerevisiae*. Scale bar indicate 100 amino acids. (**C**) MoTsc13p shares the same topology of six tramsmembrane domains as *S. cerevisiae* Tsc13p.

To identify the molecular machinery involved in nuclear degeneration in *M. oryzae*, we set out to determine whether the selective PMN pathway, described in *S. cerevisiae*, participates in degradation and recycling of nuclei during appressorium development by the rice blast fungus. In *S. cerevisiae*, *VAC8*, *TSC13* and *NVJ1* are the three important components of the PMN pathway. We interrogated the *M. oryzae* genome database using Blastp and identified putative homologues of Vac8p and Tsc13p. *VAC8* is a vacuolar membrane-associated protein, which plays important roles in several vacuolar processes in *S. cerevisiae*, including piecemeal microautophagy of the nucleus (PMN) [15], [23], [24], [25], [26]. *VAC8* was first identified in a survey of the *S. cerevisiae* genome for armadillo (ARM) repeat domain-containing proteins– conserved modules involved in mediating protein-protein interactions [23], [27], [28]. The gene was also identified independently by complementation of a class I vacuole segregation mutant, *vac8*, which contains multi-lobed vacuoles and arrests early in vacuole inheritance with defects in the cytoplasm to vacuole (Cvt) targeting pathway [29], [30]. The myristoylation of glycine and palmitoylation of three cysteine residues inside the N-terminal Src homologue 4 (SH4) domain are critical for Vac8p association with the vacuole membrane [29] and, indeed, palmitoylation at the three cysteines determines the enrichment and function of Vac8p at specific vacuolar membrane sub-domains [25], [26], [31], [32]. Vac8p interacts with different proteins through its ARM repeat domains at discrete vacuole membrane sub-domains specific to each of its distinct functions. Interacting partners include Vac17p in vacuole inheritance, Atg13 in the Cvt pathway, Nvj1p in NV junction formation and Tco89p in caffeine resistance [25], [26], [27], [31]. Homologues of *S. cerevisiae VAC8* have been reported to function in glucose-induced pexophagy in *Pichia pastoris* and in vacuolar inheritance and normal hyphal branching in *Candida albicans*, respectively [33], [34], [35], [36]. In *M. oryzae*, MoVac8p shows 85.2% identity to *S. cerevisiae* Vac8p (Figure S1 A). The predicted MoVac8p coding region has 11 putative ARM repeats and to test this prediction, we designed primers starting at the start codon predicted in the genome database and performed 3′ RACE. Unexpectedly, sequencing the 3′ RACE amplicon and a subsequent 5′RACE product showed that the correct start codon was 303 bp downstream of the predicted start codon within the first predicted intron (Genbank JN977613). The RADAR programme was used to align the ARM repeats of MoVac8p (http://www.ebi.ac.uk/Tools/Radar/index.html) and demonstrated that MoVac8p contains 9 ARM repeats, with repeat 8 and repeat 9 interrupted by 53 amino acids, which contrasts significantly with the 11 continuous ARM repeats in *S. cerevisiae* Vac8p (Figure 2). MoVac8p shares similar N-terminal acylation sites to those found in *S. cerevisiae* Vac8p (Figure 2), consistent with its predicted function.

A second major component of the PMN pathway in yeast, the *TSC13* gene, encodes enoyl reductase, an enzyme that catalyzes the last step of long-chain fatty acid (C_16_ and C_18_) elongation to produce very-long-chain fatty acids (VLCFAs) [37]. Tsc13p is an integral membrane protein located in the peripheral and peri-nuclear endoplasmic reticulum (ER), enriched at NV junctions, and is essential for cell viability [15], [22], [37]. The activity of Tsc13p in the VLCFA elongation cycle has been proposed to contribute to the biogenesis of PMN blebs [22]. There have, however, been no reports of the functions of *VAC8* or *TSC13* orthologues in any filamentous fungus to date. The *M. oryzae* MoTsc13p showed 60.1% identity to *S. cerevisiae* Tsc13p, with six predicted transmembrane domains (Figure 2 C), consistent with the topology of *S. cerevisiae* Tsc13p [38]. MoTsc13p has conserved amino acids (Figure S1 B), essential for activity of Tsc13p [38] (Genbank JN977614). Importantly, we were unable to find a homologue of *S. cerevisiae NVJ1*, using either nucleotide or amino acid sequences of *NVJ1*, based on BLASTP or TBLASTN analysis, in the *M. oryzae* genome database, or by immuno-precipitation which we used to identify proteins interacting with MoVac8-GFP (data not shown).

### MoVac8-GFP localises to the vacuole membrane and Tsc13-GFP to the perinuclear and peripheral ER membrane

**Figure 3:**
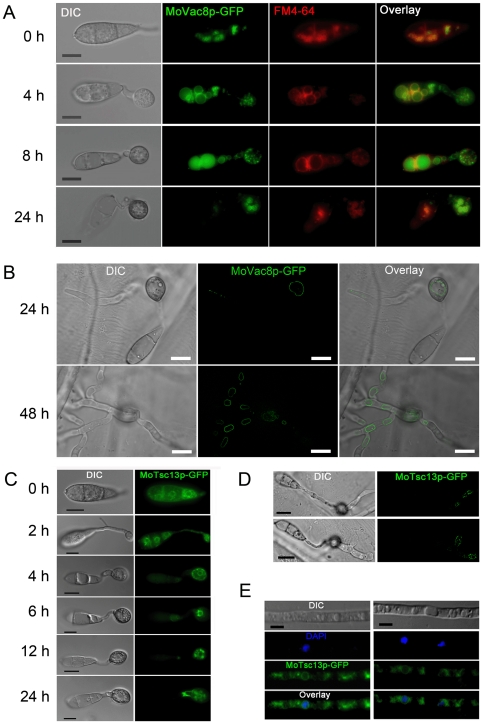
Sub-cellular localisation of MoVac8p and MoTsc13p in *Magnaporthe oryzae*. (**A**) MoVac8p-GFP is localised at the vacuolar membrane in both conidia and appressoria. FM4–64 was used to stain vacuoles and endosomes of Guy11, expressing *MoVAC8*:*GFP*. Conidia were collected and resuspended in 50 µl of CM with 7.5 µM FM4–64. Appressorium development was observed on coverslips at indicated time points. (**B**) MoVac8p-GFP is localised on the vacuolar membrane in penetration pegs and invasive hyphae. Penetration of onion epidermis was examined at indicated time points. (**C**) Tsc13p-GFP is associated with of perinuclear and peripheral ER in both conidium and appressorium. (**D**) Tsc13p-GFP is associated with perinuclear and peripheral ER in penetration pegs and invasive hyphae. Images were taken from onion epidermis infected with Guy11 expressing *MoTSC13:GFP* at 24 hpi. (**E**) MoTsc13p-GFP is localised at the perinuclear and peripheral ER in hyphae grown in CM. DAPI was used to stain nuclei of Guy11 expressing *MoTSC13*:*GFP*. Scale bar = 10 µm.

**Figure 4:**
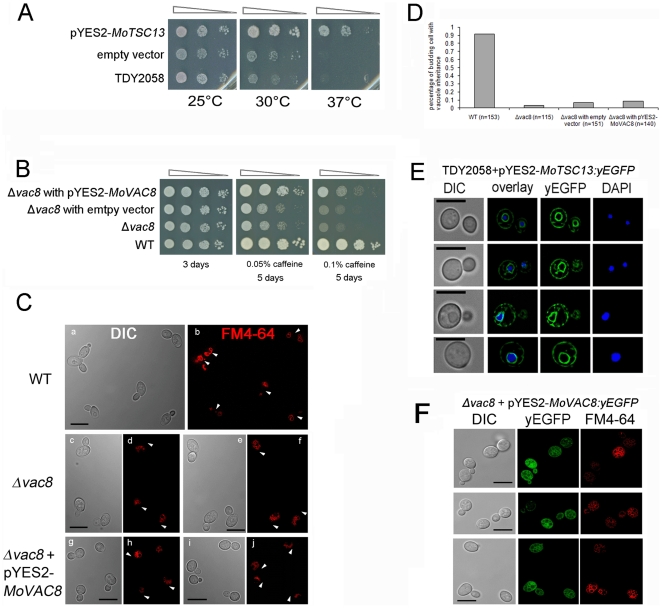
*MoTSC13* is functionally equivalent to *S. cerevisiae TSC13*, while *MoVAC8* only partially substitutes for *VAC8*. (**A**) *MoTSC13* complemented the temperature-sensitive lethality of the yeast strain TDY2058 (double mutant *tsc13-1 Δelo*). Yeast *tsc13-1 Δelo* mutants were transformed either with empty vector or a plasmid expressing *MoTSC13* cDNA under control of the galactose-inducible *GAL1* promoter. Cells were grown in YPDA overnight, normalized to and subjected to 10-fold serial dilutions, spotted onto SD+Gal plates and incubated at 26°C, 30°C or 37°C for 3 days prior to photographing. The experiments were carried out in triplicate, examining two independent yeast transformants. (**B**) *MoVAC8* complemented the caffeine sensitivity of yeast strain BY4741 *vac8*Δ*::KANMX4*. Yeast *vac8*Δ*::KANMX4* mutants were transformed with empty vector or a plasmid expressing *MoVAC8* cDNA under the control of the *GAL1* promoter. Cells were grown in YPDA overnight, normalized and subjected to 10-fold serial dilutions, spotted onto SD+Gal plates containing either 0.05% or 0.1% caffeine, and incubated at 26°C for the indicated period of time. The experiments were carried out in triplicate, examining two independent yeast transformants. (**C**) *MoVAC8* does not complement vacuole morphology and vacuole inheritance defects of yeast mutant *vac8*Δ*::KANMX4*. FM4–64 was used to stain cells of *S. cerevisiae*, *vac8Δ::KANMX4* and *vac8Δ::KANMX4* strain, expressing *MoVAC8* by pulse-chase labelling. Arrows indicates presence (b) or absence (d, f, h, j) of segregating vacuoles. (**D**) *MoVAC8* failed to complement vacuole inheritance defects of yeast mutant *vac8*Δ*::KANMX4* during budding. Pulse-chase labelled *S. cerevisiae* cells by FM4–64 were counted for the proportion of daughter cells carrying vacuoles inherited from the mother cell during budding. (N indicates the total number of cells counted). (**E**) MoTSC13p localises to the perinuclear and peripheral ER membrane in *S. cerevisiae*. yEGFP tagged MoTSC13p was expressed in yeast strain TDY2058 under control of the *GAL1* promoter in plasmid pYES2. Cells were stained with DAPI to visualise nuclei. (**F**) MoVAC8p failed to accumulate at the vacuolar membrane in *S. cerevisiae*. yEGFP tagged MoVAC8p was expressed in yeast strain *vac8*Δ*::KANMX4* under control of *GAL1p* promoter in plasmid pYES2.

To investigate whether MoVac8p and MoTsc13p showed similar sub-cellular localisation patterns to their yeast counterparts (consistent with a PMN function) we generated *MoVAC8*:*GFP* and *MoTSC13*:*GFP* gene fusion constructs and expressed them under their native promoters in the wild type *M. oryzae* strain Guy11. MoVac8-GFP showed a membrane-associated distribution pattern in conidia, appressoria and invasive hyphae, as shown in Figure 3 A and B. When stained with FM4–64 during appressorium development, MoVac8-GFP showed a similar distribution to FM4–64 (Figure 3A), suggesting that MoVac8p in *M. oryzae* localises to the vacuolar membrane and partially to the vacuolar lumen. Vacuoles in the conidium were initially small and those inside the apical cell moved into the germ tube and nascent appressorium (Figure 3 A; 4 h timepoint), after which all vacuoles in the conidium fused together to form a large central vacuole (Figure 3 A; 4 h and 8 h timepoints). The vacuole finally degenerated in the conidium (Figure 3 A) after 24 h and eventually MoVac8:GFP disappeared from the collapsing conidium following appressorium development. The mature appressorium contained a large central vacuole after 24 h (Figure 3 B), consistent with previous studies showing the importance of the vacuole as a key lytic organelle in degrading lipid storage reserves during appressorium development in *M. oryzae* [39].

In hyphae of transformants expressing *MoTSC13*:*GFP*, the fusion protein was also membrane-associated, as shown in Figure 3E. To stain nuclei, 2,4,-Diamidino-phenyl-indole (DAPI) was used in hyphae of these transformants and showed that the MoTsc13p is detected predominantly at locations consistent with the peri-nuclear ER membrane and peripheral ER (Figure 3E). This membrane-associated distribution pattern of MoTsc13p was also found in the conidium, appressorium, penetration peg and invasive hyphae during plant infection (Figure 3 C and D). During appressorium development, MoTsc13p was detected in the germ tube and differentiating appressorium, indicating that MoTsc13p-anchored ER moves into the appressorium (Figure 3C). Taken together, these data revealed that both MoVac8p and MoTsc13p showed sub-cellular distribution patterns consistent with a role in a variety of vacuole and ER functions.

### Conservation of *MoVAC8* and *MoTSC13* function

To determine whether *MoVAC8* and *MoTSC13* are functional equivalents of *S. cerevisiae VAC8* and *TSC13*, respectively, complementation experiments were performed. Heterologous expression of a *MoTSC13* cDNA in a *S. cerevisiae tsc13-1* Δ*elo* double mutant, was sufficient to restore its ability to grow at 37°C, as shown in Figure 4 A, suggesting that *MoTSC13* is the functional homologue of yeast *TSC13* enoyl-CoA reductase [40], [41]. When we expressed yeast enhanced GFP (yEGFP)-tagged *MoTSC13* in *tsc13-1* Δ*elo* mutants, they also complemented the mutant phenotype and displayed the same perinuclear and peripheral ER membrane-anchoring distribution in yeast cells (Figure 4E). We conclude that *MoTSC13* probably serves an evolutionarily conserved function in catalyzing the fourth reaction of fatty acid elongation to produce VLCFAs in both fungi [37].

In *S. cerevisiae*, Δ*vac8* mutants show various vacuole-associated phenotypes, including caffeine hypersensitivity, multi-lobed vacuoles, loss of protein transport from the cytoplasm to vacuoles, an inability of budding daughter cells to inherit vacuoles from the mother cell and, importantly defects in PMN [15], [23], [24], [25], [26]. When *MoVAC8* cDNA was expressed in a *S. cerevisiae* Δ*vac8* mutant BY4741 under control of the *GAL1* promoter, growth of yeast was partially restored in the presence of 0.05% or 0.1% caffeine (Figure 4 B), suggesting that *VAC8* has conserved functions between *S. cerevisiae* and *M. oryzae* in regulating the caffeine response. We used pulse-chase labelling with FM4–64 to track vacuolar morphology and inheritance during budding of the *S. cerevisiae* strain BY4741 expressing *MoVAC8*. Vacuoles remained multi-lobed and identical to those observed in the Δ*vac8* mutant. Moreover, yeast daughter cells failed to inherit vacuoles from mother cells (Figure 4C and D). To test whether MoVac8p was targeted to the vacuolar membrane of *S. cerevisiae*, a *MoVAC8 cDNA:yEGFP* was constructed and introduced into the *S. cerevisiae* Δ*vac8* mutant BY4741. Interestingly, MoVac8p was mostly distributed in the cytoplasm and failed to accumulate at the vacuolar membrane (Figure 4F), indicating that the N-terminal vacuole-membrane anchoring peptide found in *M. oryzae* is not fully functional in *S. cerevisiae*. Partial complementation of the yeast Δ*vac8* mutant by *MoVAC8* may reflect the different structural organization of Vac8p between *S. cerevisiae* and *M. oryzae*. Taken together, we conclude that *MoTSC13* is a direct functional homologue of *S. cerevisiae TSC13* while *MoVAC8* appears to fulfil a role in the caffeine response but may have diverged in both structure and function in *M. oryzae*.

### *MoVAC8* and *MoTSC13* are not required for conidial nuclear degeneration during appressorium development

**Figure 5:**
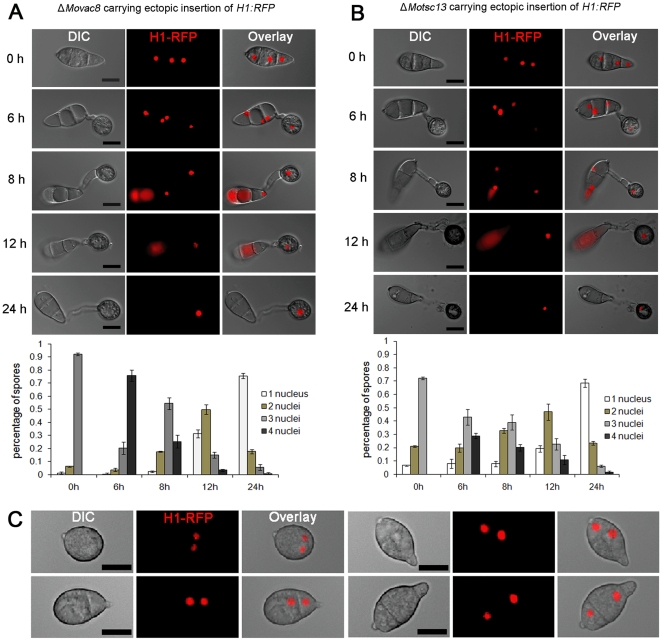
*MoVAC8* and *MoTSC13* are not required for nuclear degeneration in *M. oryzae*. (**A**) Nuclear degeneration during appressorium development occurs independently of *MoVAC8*. Upper panel: time course live cell images showing nuclear division and nuclear degeneration during appressorium development in *M. oryzae* Δ*Movac8* deletion mutants. Δ*Movac8* expressing *H1:RFP* were examined by epifluorescence microscopy at indicated time points during appressorium development. Lower panel: time series of bar charts showing the percentage of spore germlings of Δ*Movac8* containing between 0 and 4 nuclei (mean ± SD, n>100, triple replications). (**B**) Nuclear degeneration during appressorium development occurs independently of *MoTSC13*. Upper panel: time course live cell images showing nuclear division and nuclear degeneration during appressorium development in *M. oryzae MoTSC13* deletion mutants. Δ*Motsc13* expressing *H1:RFP* were examined by epifluorescence microscopy at indicated time points during appressorium development. Lower panel: time series of bar charts showing the percentage of spores in Δ*Motsc13* containing between 0 and 4 nuclei (mean ± SD, n>100, triple replications). (**C**) Micrographs showing abnormal conidia of Δ*Motsc13* containing two nuclei. Scale bar = 10 µm.

To determine the function of both putative PMN proteins in *M. oryzae*, we generated Δ*Movac8* and Δ*Motsc13* mutants in Guy11 using a split marker method and confirmed targeted gene deletion by Southern blot hybridization (Figure S2). In order to determine whether *MoVAC8* and *MoTSC13* are involved in nuclear degeneration, *H1*:*RFP* was introduced into both Δ*Movac8* and Δ*Motsc13* mutants to allow live cell imaging of nuclear behaviour. We monitored nuclear numbers during appressorium development and, strikingly, nuclei showed the same behaviour between Δ*Movac8*, Δ*Motsc13* and Guy11, as shown in Figure 5A and B. These observations suggest that nuclear degeneration occurs independently of a PMN pathway in *M. oryzae* because nuclear degeneration was unaffected in either mutant. However, compared to Guy11 and Δ*Movac8*, a much higher percentage (∼30%) of conidia of the Δ*Motsc13* mutant contained only one or two nuclei, as a consequence of a conidial morphology phenotype that was associated with loss of *MoTSC13* (Figure 5C).

**Figure 6:**
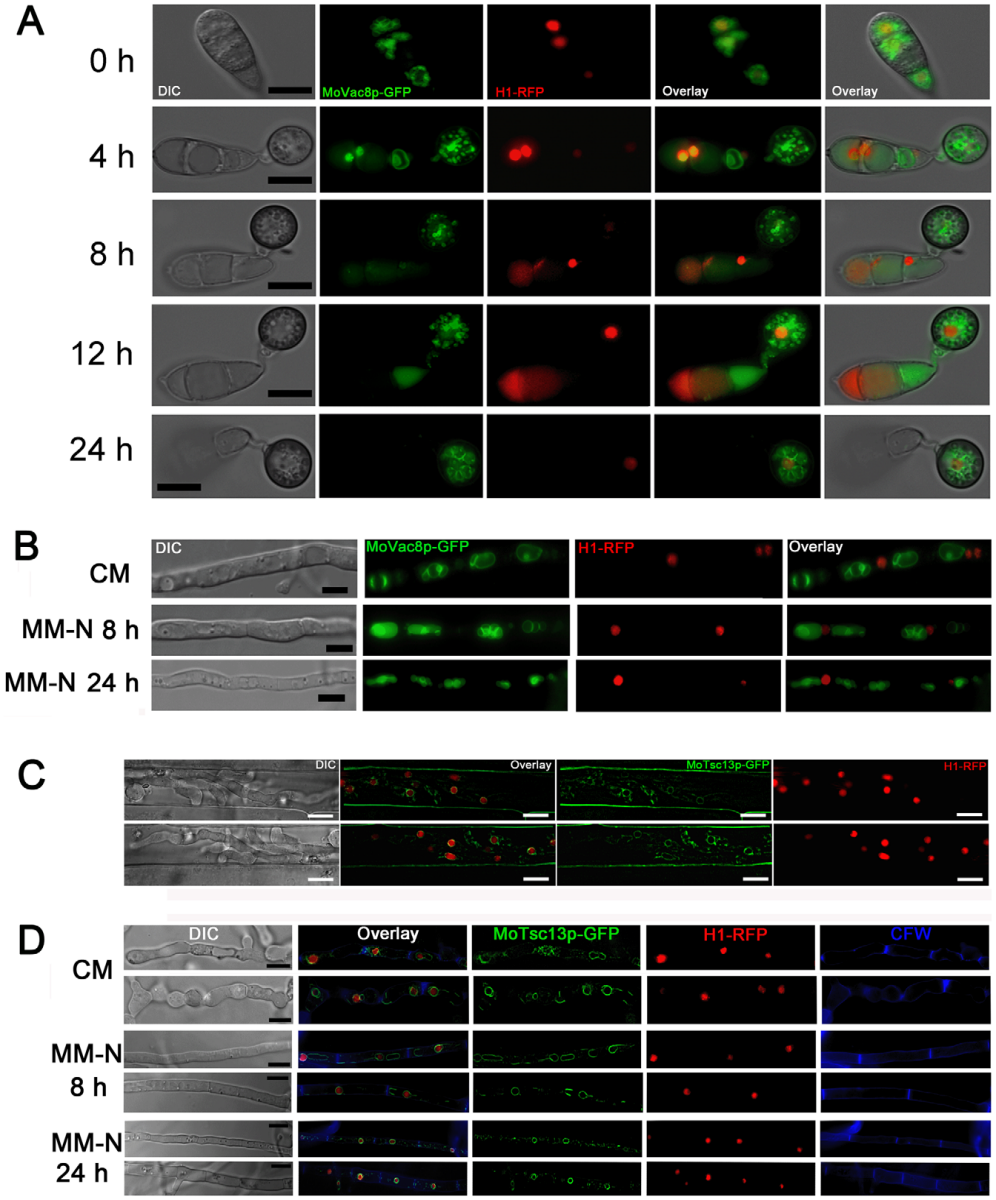
Nuclear-vacuolar (NV) junctions are not present in *M. oryzae* during appressorium development or starvation stress. (**A**) Time course live cell imaging of appressorium development in *M. oryzae* expressing both *MoVAC:GFP* and *H1:RFP*. (**B**) Micrographs of hyphae of *M. oryzae* expressing both *MoVAC:GFP* and *H1:RFP*. *M. oryzae* was grown in nutrient-rich medium (CM) or nitrogen stress minimal medium (MM-N) for the indicated period of times, and hyphae collected for epifluorescence microscopy. Hyphae cultured in CM for 40 h were washed with distilled water, before being transferred into MM-N. (**C**) MoTsc13p is associated with perinuclear and peripheral ER membrane in invasive hyphae. *M. oryzae* expressing both *MoTSC13:GFP* and *H1:RFP* was inoculated onto rice sheath epidermis for 36 h before epifluorescence microscopy. (**D**) Distribution of MoTsc13p-GFP is not affected by environmental nutritional status. Hyphae of *M. oryzae* expressing both *MoTSC13:GFP* and *H1:RFP* were grown in CM or MM-N for epifluorescence microscopy. Pictures shown are representatives of at least 30 different hyphae cell compartments analyzed for each time point. Scale bar = 10 µm.

We also introduced *MoVAC8*:*GFP* gene fusion into the *H1*:*RFP*-expressing strain of *M. oryzae* to investigate formation of putative nuclear-vacuolar (NV) junctions. A time-course experiment was carried out with the *MoVAC8*:*GFP*, *H1*:*RFP* strain to observe appressorium development (Figure 6 A). The large vacuole in the conidium occupied the majority of the conidial cell volume and, consequently, the nucleus and vacuole were often apposed to one another, but no distinct, regulated physical interaction of vacuoles and nuclei was observed. The typical teardrop-shaped blebs, which in *S. cerevisiae* originate from NV junctions and release PMN vesicles into the vacuole [15], [16], were also absent from conidia undergoing autophagic cell death, as shown in Figure 6A. More importantly, Vac8p was degraded in the conidium at a time when nuclei were still present (Figure 6 A; 4 h and 8 h timepoints), indicating that vacuole degeneration may proceed before the onset of nuclear degeneration. Because PMN is induced to high levels in *S. cerevisiae* by starvation [15], [16], we also carried out microscopy of the *MoVAC8*:*GFP*; *H1*:*RFP* strain grown under nitrogen starvation conditions to determine whether NV junctions were apparent after starvation of *M. oryzae*. No obvious NV junctions or teardrop-shaped blebs were detected in *M. oryzae* following starvation stress, as shown in Figure 6B.

In comparison to the accumulation of *S. cerevisiae* Tsc13p at NV junctions from peripheral and nuclear ER pools during starvation stress [22], [37], we found that MoTsc13-GFP in the conidium of *M. oryzae* was equally distributed at perinuclear and peripheral ER membranes during appressorium development (Figure 3C). When the *MoTSC13*:*GFP* gene fusion was introduced into Guy11 carrying *H1*:*RFP*, MoTsc13p also showed a nuclear and peripheral ER membrane-associated distribution pattern (Figure 6 C), and no enrichment of the MoTsc13p at the nuclear membrane was observed in hyphae grown under starvation conditions (Figure 6 D). Taken together, we conclude that there is no formation of NV junctions, the typical structures of PMN, in *M. oryzae* either during appressorium development or following nitrogen starvation.

### The N-terminal SH4 domain of MoVac8p is required for association of MoVac8p with the vacuolar membrane

The N-terminus of Vac8p in *S. cerevisae* contains a SH4 domain, which serves as a membrane anchoring signal peptide [31]. SH4 domains are normally composed of 18 amino acids and characterised by a myristoylation motif (MGxxxS/Tx) and a palmitoylation site (a cysteine residue) or several basic amino acids [32]. The SH4 domain within Vac8p of *S. cerevisae* for instance, possesses three palmitoylation sites, which play roles in the localisation of Vac8p in the vacuolar membrane [26], [31]. Analysis of the N-terminal sequences of MoVac8p revealed the presence of a myristoylation motif and three potential palmitoylation sites, as shown in Figure 2 A.

**Figure 7:**
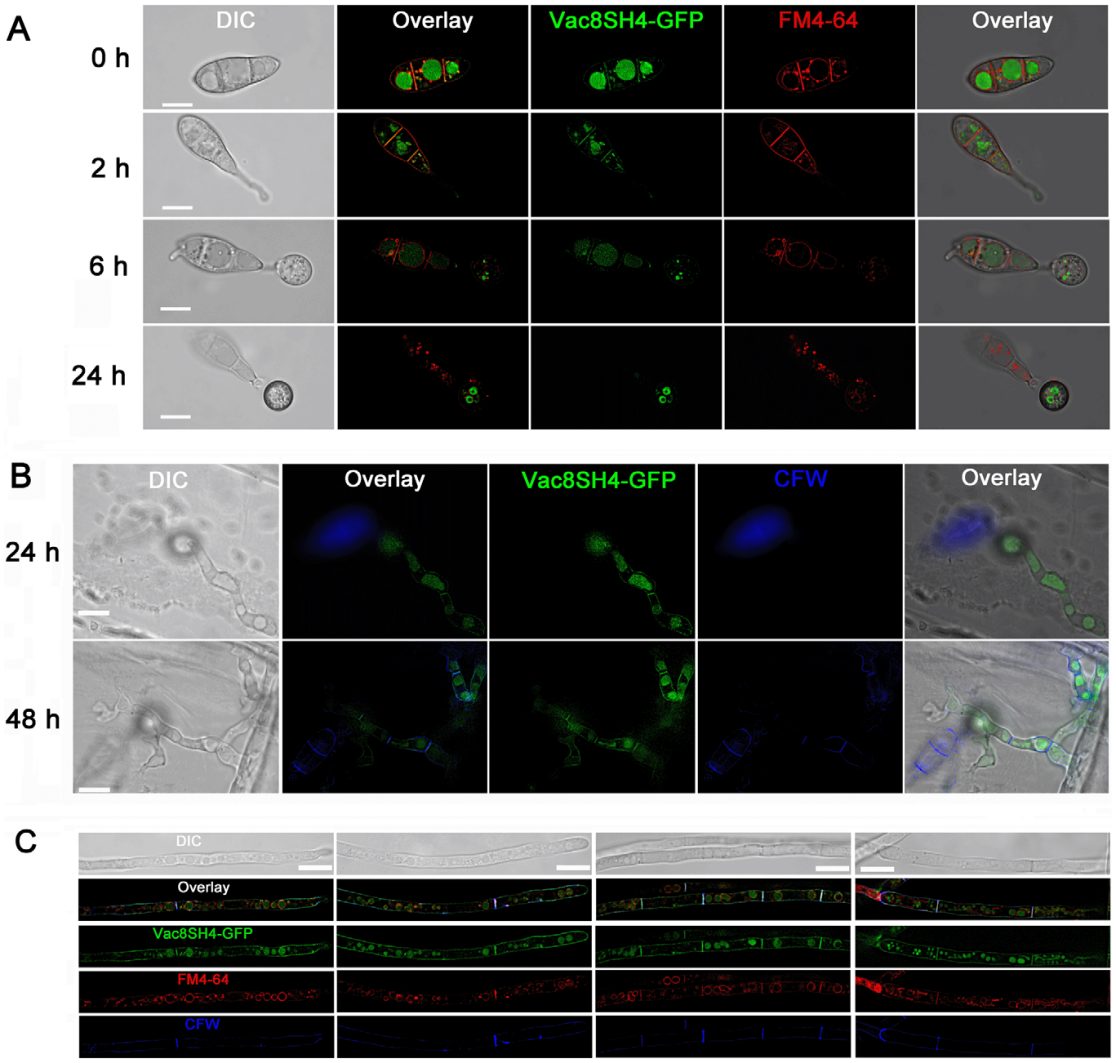
The N-terminus of MoVAC8p contains a SH4 domain. (**A**) SH4:GFP fusion proteins are anchored at the vacuole, septa and the plasma membrane in both conidia and appressoria. The first 21 amino acid residues of MoVAC8p were fused into the N-terminus of GFP to produce SH4:GFP and subcellular localisation of the GFP fusion analysed by epifluorescence microscopy. Guy11 conidia expressing SH4:GFP fusion proteins were stained with FM4–64 and allowed to germinate and undergo appressorium development. SH4:GFP fusions colocalise with the FM4–64-stained vacuole, septa and plasma membrane. (**B**) SH4:GFP fusion proteins associate with the vacuole, septa and the plasma membrane in penetration pegs and invasive hyphae. Penetration of onion epidermis was examined at indicated time points and Calcofluor White (CFW) used to stain the cell wall. SH4:GFP fusion proteins colocalise with the vacuole (as seen in the bright field image) and CFW-stained cell wall. (**C**) SH4:GFP fusion proteins were targeted to the vacuole, septa and the plasma membrane in vegetative hyphae. Vegetative hyphae expressing SH4:GFP fusion proteins were grown in CM for 24 h, and 200 µl of the cultures incubated with 7.5 µM FM4–64 at 26°C for 1 h, followed by CFW staining. In vegetative hyphae, SH4:GFP fusion proteins also colocalise with FM4–64- stained vacuoles and CFW-stained septa and cell walls. Scale bar = 10 µm.

To investigate whether MoVac8p contains a functional SH4 domain, we used the first 21 amino acids of MoVac8p to generate a putative SH4 domain:GFP fusion protein. Localisation of the SH4 domain:GFP fusion protein was examined in conidia, appressoria, invasive hyphae and vegetative hyphae by epifluorescence microscopy (Figure 7). The distribution of the SH4 domain:GFP fusion protein in each cell type was coincident with FM4–64 stained membranes and vacuoles, and also overlapped with the CFW stained cell wall and septa, suggesting that SH4:GFP is membrane-associated.

To address whether the myristoylation and palmitoylation sites of the putative SH4 domain are involved in localisation of MoVac8p at the vacuolar membrane, we performed site-directed mutagenesis to generate constructs expressing variants of MoVac8p-GFP, in which glycine and cysteine residues within the SH4 domain were replaced by alanine residues (Figure S3A). Single point mutations of *MoVAC8:GFP*, including *G2A*, *C4A*, *C8A* and *C9A*, did not abolish association of MoVac8p-GFP with the vacuolar membrane (See Figure S3B and Figure S4). However, these single point mutations did result in mislocalisation of MoVac8p-GFP into the septal pore region in vegetative hyphae (Figure S3B and C), but not in conidia (Figure S4, at least 50 conidia were examined for each variants). When two of the palmitoylation sites were mutated, including *C4A/C8A*, *C4A/C9A* and *C8A/C9A*, mislocalisation of MoVac8p-GFP in the mycelia septa pore area was further increased (Figure S3B and C), and MoVac8p-GFP in the *C4A/C8A*, *C4A/C9A* variants showed strong cytosolic localisation and loss of association with the vacuolar membrane. The *C8A/C9A* substitution resulted in an increase in the mislocalisation into the septa pore (Figure S3 B and C). Similar results were obtained in mutants expressing the double point mutations, *C4A/C8A*, *C4A/C9A* and *C8A/C9A* when conidia were examined (Figure S4). Moreover, in vegetative hyphae and conidia of the MoVac8p-GFP strain expressing a triple point mutation *C4A/C8A/C9A*, the association of fusion proteins with the vacuolar membrane was completely disrupted resulting in completely cytosolic localisation and enrichment at the septal pore (Figure S3B and C, Figure S4). We conclude that both myristoylation and palmitoylation are involved in localisation of MoVac8p.

In *S. cerevisae* palmitoylation of Vac8 is required for caffeine resistance [26]. To examine the relationship between acylation of the SH4 domain and MoVac8p function, we therefore measured sensitivity of strains expressing mutant alleles of *MoVAC8:GFP* to caffeine. Δ*Movac8* mutants showed hypersensitivity to 0.1% caffeine, while expression of *MoVAC:GFP* restored normal growth (Figure S5). In Δ*Movac8* mutants expressing *MoVAC:GFP* variants *C4A*, *C8A*, *C9A*, *C4A/C8A*, *C4A/C9A* and *C8A/C9A*, growth on CM containing 0.1% caffeine was restored, but variant *G2A* only partially restored growth and the triple mutant *C4A/C8A/C9A* failed to restore full growth (Figure S5). These results indicate that myristoylation, in particular, and complete palmitoylation of MoVac8p plays a role in caffeine resistance in *M. oryzae*.

### *MoVAC8* is necessary for the caffeine response, while *MoTSC13* is required for full virulence and cell wall integrity

**Figure 8:**
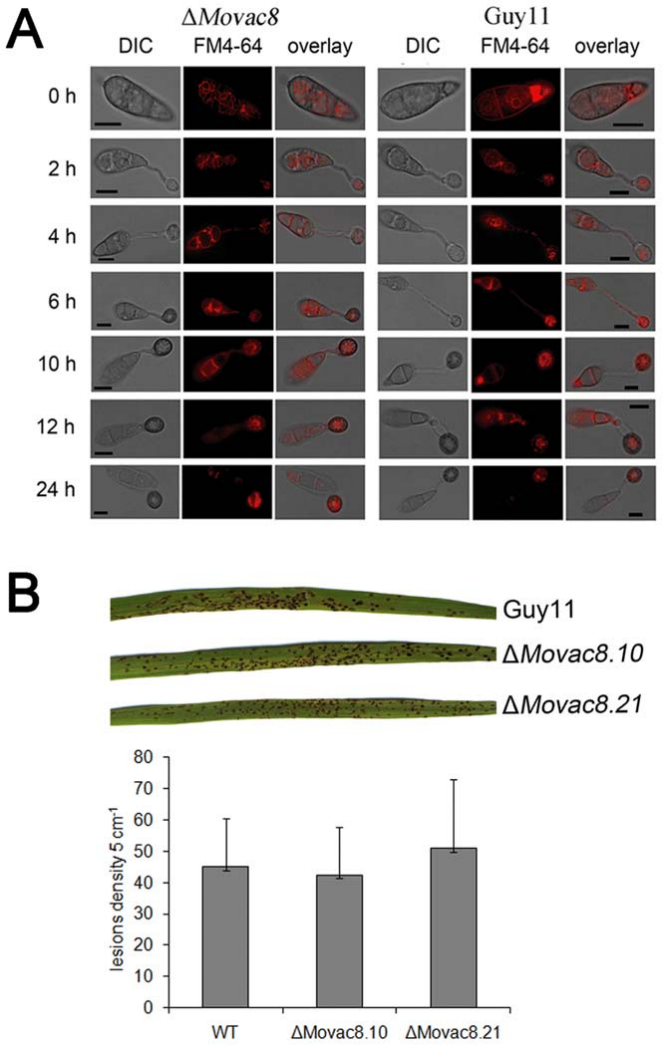
Phenotypic analysis of Δ*Movac8* mutants. (**A**) *MoVAC8* is not involved in vacuole movement from the conidium into the appressorium. FM4–64 was used to stain endosomes and vacuoles in conidia to visualize movement of vacuoles during appressorium development. (**B**) Rice blast infection assay of Δ*Movac8* mutants on rice leaves. The virulence of Δ*Movac8* is similar to that of strain Guy11. Lesion density represents the lesion number per 5 cm of infected leaf area on rice CO-39 seedlings (n>40).

**Figure 9:**
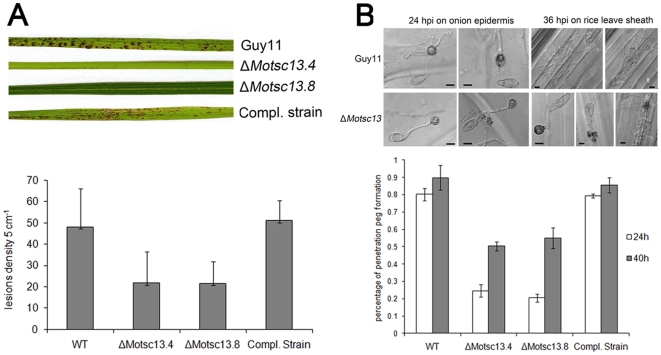
*MoTSC13* is required for full virulence of *M. oryzae*. (**A**) Targeted deletion of *MoTSC13* results in reduced pathogenicity on rice CO-39. Lesion density and size was severely reduced in ΔMotsc13 rice infections compared to Guy11. Full pathogenicity of Δ*Motsc13* mutants was restored by introduction of *MoTSC13:GFP* (Compl.). Lesion density represents the lesion number per 5 cm of infected leaf area (n>40). (**B**) *MoTSC13* is necessary for penetration peg formation and invasive hyphae expansion during *in planta* growth. Scale bar = 10 µm.

We investigated the functions of *MoVAC8* and *MoTSC13* by analysis of the phenotypes of each deletion mutant. In view of the role of Vac8 in vacuole inheritance and movement, we investigated movement of vacuoles and endosomes during appressorium development in both Guy11 and Δ*Movac8* mutants by staining with FM4–64. Both Δ*Movac8* mutants and Guy11 showed a similar pattern of vacuole and endosome movement, in which vacuoles in the conidium moved into the germ tube during germination and into the appressorium, during cellular differentiation (Figure 8A). Moreover, the fusion of vacuoles was not impaired in Δ*Movac8* mutants (Figure 8A). These results indicate that MoVac8p does not serve roles in vacuole inheritance or vacuole fusion during conidium germination or appressorium development. Δ*Movac8* mutants did, however, show enhanced caffeine sensitivity and slightly increased sensitivity to calcofluor white and high concentrations of Congo red, consistent with a role in cell wall integrity (Figure S7). Plant infection assays also suggested that *MoVAC8* is dispensable for pathogenicity of *M. oryzae* (Figure 9B), because Δ*Movac8* mutants caused similar numbers of disease lesions to the isogenic wild type strain Guy11 and appressoria formed normally.

In contrast to the essential function of *TSC13* in *S. cerevisiae*, *MoTSC13* is not essential for viability in *M. oryzae*, but loss of *MoTSC13* did reduce vegetative growth and conidiation of *M. oryzae* and increased sensitivity to osmotic stress and Calcofluor White (Figure S8A, B and C). Importantly, Δ*Motsc13* mutants were only able to produce very small disease lesions on rice leaves as shown in Figure 9A. Δ*Motsc13* mutants formed appressoria normally (Figure S8D), implying that neither *MoVAC8* nor *MoTSC13* serve essential functions in appressorium development. To determine which stage of infection was impaired in Δ*Motsc13* mutants, we measured appressorium turgor and the frequency of penetration peg formation (Figure 9B, Figure S8 E). Turgor was unaltered in Δ*Motsc13* mutants (Figure S8 E). However, penetration peg formation was severly impaired with only 20% of appressoria able to elaborate a penetration peg after 24 h (Figure 9B). By 36 hpi, invasive hyphae of Guy11 had moved into the second or third rice epidermal cell adjacent to the invasion site, but most Δ*Motsc13* mutant appressoria failed to penetrate, and invasive hyphae were limited to the initial cell at the invasion site (Figure 9B). Re-introduction of the *MoTSC13:GFP* fusion construct into a Δ*Motsc13* mutant restored normal vegetative growth, penetration peg formation and pathogenicity on rice leaves (Figure 9A and B; Figure S8 A). We conclude that MoTsc13 is involved in penetration hypha development during plant infection.

### Macroautophagy is required for nuclear degeneration during appressorium development

**Figure 10:**
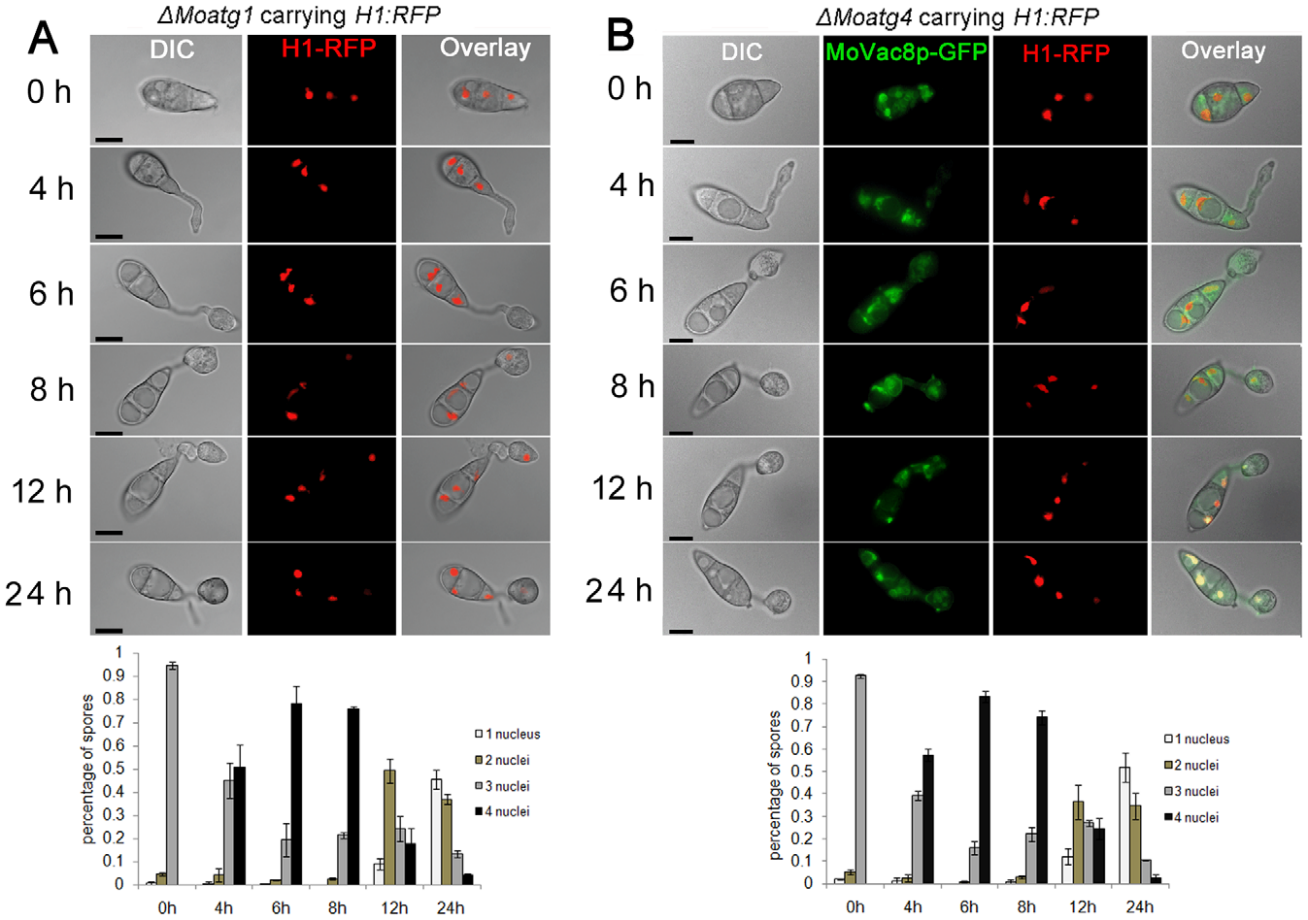
Macroautophagy is necessary for nuclear degeneration during appressorium development in *M. oryzae*. (**A**) The Macroautophagy gene *MoATG1* is required for nuclear degeneration. Upper panel: time course live cell images showing nuclear division and nuclear degeneration during appressorium development in a *M. oryzae* Δ*Moatg1* mutant. Δ*Moatg1* conidia expressing *H1:RFP* were examined by epifluorescence microscopy at indicated time points. Lower panel: time series of bar charts showing the percentage of spores in Δ*Moatg1* containing 0 to 4 nuclei (mean ± SD, n>100, triple replications). (**B**) The Macroautophagy core gene *MoATG4* is required for nuclear degeneration. Upper panel: time course live cell images showing nuclear division and nuclear degeneration during appressorium development in *M. oryzae* Δ*Moatg4* mutant. Δ*Moatg4* conidia expressing *H1:RFP* were examined by epifluorescence microscopy at indicated time points during appressorium development. Lower panel: time series of bar charts showing the percentage of spores in Δ*Moatg4* containing 0 to 4 nuclei (mean±SD, n>100, triple replications). Scale bar = 10 µm.

Given the absence of a discernable PMN pathway, we decided to investigate alternative means by which nuclei might be degraded in *M. oryzae*. We first expressed the *H1*:*RFP* gene fusion in a Δ*Moatg1* mutant to allow *in vivo* observation of nuclei during appressorium development in a macroautophagy-deficient mutant. Live cell imaging showed that nuclei in the conidium were misshapen and failed to degenerate even after 24 hpi, as shown in Figure 10A. We also examined whether *MoATG4* was required for nuclear degeneration in *M. oryzae*. To achieve this, we performed targeted gene deletion of *MoATG4* in a *M. oryzae* strain expressing both *MoVAC8*:*GFP* and *H1*:*RFP* (Figure S2 C). We found that *MoATG4* was required for conidial collapse and nuclear degeneration during appressorium development (Figure 10B). We went on to examine nuclear degradation in targeted deletion mutants affecting both macro-autophagy and selected autophagy. We found that mutants in genes associated macro-autophay all showed defects in nuclear degeneration as observed in Δ*Moatg1* and Δ*Moatg4* mutants [11]. By contrast mutants in genes associated exclusively with selective autophagy (*ATG11*, *ATG24*, *ATG26*, *ATG27*, *ATG28*, *ATG29*) did not show any defect in nuclear degeneration (data not shown).

We also investigated the localisation of *MoTSC13*:*GFP* and *MoVAC8*:*GFP* gene fusion constructs in a Δ*Moatg1* mutant in order to see the effect of arresting autophagy on protein localisation during infection related development We observed that MoTsc13p-GFP accumulated in the conidium until 24 hpi (Figure S6), in contrast to the gradual disappearance of MoTsc13p in Guy11 after 4–6 h (Figure 3C). In Guy11 expressing *MoVAC8*:*GFP*, vacuole degeneration started after completion of mitosis, and vacuoles were absent from the conidium after 24 hpi (Figure 3A). While in an Δ*Moatg1* mutant expressing *MoVAC8*:*GFP*, vacuoles failed to degenerate even after 24 hpi (Figure S6), suggesting a crucial role for macroautophagy in mediating vacuole degeneration or trafficking from the conidium during appressorium development in *M. oryzae*. Consistent with this idea, vacuoles also accumulated in the conidium of Δ*Moatg4* mutants, as shown in Figure 10B. When considered together these data suggest that macroautophagy is important for nuclear degeneration, ER degeneration and vacuole degeneration within conidia during plant infection by *M. oryzae*.

### Conclusions

In this study we set out to determine the mechanism by which nuclei are broken down in conidia of the rice blast fungus prior to appressorium formation. Appressorium-mediated plant infection by the rice blast fungus is tightly linked to cell cycle control and conidial cell death and degeneration of nuclei within the spore is an essential pre-requisite to successful plant infection [7], [10], [11]. In yeast, it is apparent that nuclei are degraded by a selective autophagic process, PMN, in which nuclei bind to vacuoles via nucleus-vacuole (NV) junctions. These NV junctions invaginate and release PMN vesicles containing nuclear material into the lumen of vacuoles for hydrolysis [16], [17]. We have demonstrated that *M. oryzae* possesses two strong candidate PMN genes, *MoVAC8* and *MoTSC13*, but does not possess a *NVJ1* homologue and, importantly, does not appear to form NV junctions associated with PMN-mediated nuclear breakdown. Furthermore, we have shown that mutants lacking either *MoVAC8* and *MoTSC13* still undergo nuclear breakdown and appressorium differentiation, indicating that PMN does not mediate nuclear degeneration in *M. oryzae*.

Based on yeast complementation experiments, we observed that *MoVAC8* fulfils only a sub-set of the functions of its yeast counterpart and was unable to localize correctly when expressed in a yeast Δ*vac8* mutant. This is likely to be a consequence of its distinct structure with only 9 ARM repeats present in the protein, compared to 11 in Vac8p. It is clear, however, that MoVac8 is a vacuolar membrane protein, which is both myristoylated and palmitoylated [42] in *M. oryzae* and is involved in the response to caffeine, because *ΔMovac8* mutants show hypersensitivity to caffeine (1,3,7-trimethyl xanthine). This function is also conserved when *MoVAC8* was expressed in a yeast Δ*vac8* mutant. Caffeine sensitivity in *S.cerevisiae* appears to be associated with the Pkc1/cell integrity pathway because caffeine treatment induces rapid phosphorylation of the Mpk1 MAP kinase and leads to large scale changes in gene expression associated with cell wall stress [43]. However, the similarity in transcriptional response to rapamycin treatment, coupled with the hypersensitivity of Tor1 kinase mutants to caffeine, also point to an effect on the Ras/cAMP response pathway, and the control of cellular viability, which is coupled with the regulation of autophagy. The hypersensitivity of Δ*Movac8* mutants to caffeine may therefore be associated with an impairment in vacuole transport function, which is consistent with the requirement for myristoylation and palmitoylation for vacuolar membrane localization. Interestingly, the wider reported roles for Vac8p in vacuolar inheritance were not conserved in *M. oryzae*, suggesting significant divergence in function, consistent with the distinct structural organisation of the protein. The *MoVac8-GFP* gene fusion did allow visualization of vacuole behaviour during infection-related development in *M. oryzae* in live cell imaging experiments and highlighted the importance of formation of a large central appressorial vacuole during during appressorium turgor generation, which had been suggested in earlier cytochemical studies [39]. We can therefore conclude that MoVac8 is a vacuolar protein that is unlikely to serve a role in PMN in the rice blast fungus, but instead plays a role in vacuolar function which may be vital for contending with abiotic stresses such as exposure to caffeine.

In contrast to *MoVAC8*, *MoTSC13* appears to have a highly conserved function as an enoyl reductase that catalyzes the fourth reaction of fatty acid elongation to produce very long chain fatty acids. This function appears to be completely conserved with the role of TSC13p in yeast, but strikingly, *MoTSC13* is not essential for cellular viability in *M. oryzae* and Δ*Motsc13* mutants instead grow well in culture. Furthermore, we found no evidence for a role for MoTsc13p in PMN and there was no distinct localization of the protein at specific NV junctions. Instead, we found that MoTsc13-GFP localized to the perinuclear and peripheral ER. Importantly, we did observe that Δ*Motsc13* mutants are significantly impaired in their ability to cause rice blast disease and that this results as a consequence of a reduced ability to colonize rice epidermal cells following appressorium-mediated penetration of the cuticle. We can conclude that very long chain fatty acid biosynthesis is therefore likely to be important in invasive hyphae development, perhaps pointing to the membrane components of invasive hyphae possessing a distinct lipidic characteristic compared to those of vegetative hyphae– a feature worthy of future investigation.

The final conclusion that can be made from this study is that nuclear degeneration during appressorium formation, which is known to be essential for plant infection [7], [10], [11], occurs via non-selective macroautophagy. In contrast to yeast, there is no evidence for a separate selective PMN process in *M. oryzae*. We found that macro-autophagy-associated genes such as *MoATG1* or *MoATG4* were necessary for nuclear degeneration and their absence rendered the fungus non-pathogenic [11], whereas mutations in genes affecting selective forms of autophagy did not show any difference from the wild type Guy11. Macroautophagy has very recently been reported to mediate nuclear degeneration in *Aspergillus oryzae* [44], but in that case involved formation of large ring-like autophagosomal structures (1–2 µm) that encircled and mediated degradation of whole nuclei in *A. oryzae* basal cells. In this study we were only able to detect punctate autophagosomes in both conidia and appressoria of *M. oryzae* (consistent with [11], [12], [13]), rather than the much larger, ring-like autophagosome structures reported in *A. oryzae* [44], suggesting that nuclear breakdown may be performed by distinct macroautophagy-dependent processes in filamentous fungi. Furthermore, nuclei did not appear to be degraded in their entirety, but rather there was dissolution of nuclear material, which could be observed both sytoplasmically and ithin vacuoles during autophagy. When considered together, we can conclude that conidial cell death and nuclear degeneration, which occur as part of the essential programme for appressorium-mediated plant infection by *M. oryzae*, both require non-selective autophagy, which re-cycles the contents of these cells, including nuclei, ER and other organelles into the specialized infection structure, prior to plant cuticle rupture and tissue colonization.

## Materials and Methods

### Fungal strains, growth conditions, and DNA analysis

The fertile rice pathogenic *M. oryzae* strain, Guy11, was used in all studies [45]. Culture, maintenance, and storage of *M. oryzae* isolates, media composition, nucleic acid extraction, and fungal transformation were all as previously described [46]. Yeast strains were manipulated using standard methods. All primers used in this study are described in Table S1. *S. cerevisiae* strain BY4741 *vac8*Δ*::KANMX4* (*MAT*a *his3D1 leu2D0 met15D0 ura3D0 vac8*Δ*::KANMX4*) used for expression of *MoVAC8* cDNA was obtained from EUROSCARF. *S. cerevisiae* strain TDY2058 (*MAT*α *elo3*::*TRP1 tsc13-1 ade2-101 ura3-52 trp1*D *leu2*D) used for expression of *MoTSC13* cDNA was kindly provided by Dr. Teresa M. Dunn (Department of Biochemistry, Uniformed Services University of the Health Sciences, Bethesda, Maryland). Gel electrophoresis, restriction enzyme digestion, gel blots, PCR and sequencing were performed using standard procedures [47].

### Targeted deletion of *MoVAC8*, *MoVAC8:GFP* and *MoVAC8SH4:GFP* fusion plasmid construction, site-directed mutagenesis of *MoVAC8:GFP* and Δ*Movac8* complementation

The split-marker recombination method was used for efficient targeted deletion of *M. oryzae* genes [11], [48]. The *hph* gene, which confers resistance to HygromycinB (HYG) was used as the split marker. The two split *hph* templates were amplified by primers M13F with HYsplit and M13R with YGsplit, as previously described [11]. A 1 kb sequence flanking either side of the *MoVAC8* coding sequence was amplified, with left flanking (LF) sequences amplified by primers vac850.1 and vac8m13f, right flanking (RF) sequences amplified with primers vac830.1 and vac8m13r. The LF sequences were fused with split HY, using primers vac850.1 and HYsplit to form LF-HY, while the RF sequences fused with the split YG fragment using primers YGsplit and vac830.1, to form YG-RF. The resulting amplicons LF-HY and YG-RF were gel-purified and co-transformed into protoplasts of Guy11. The Δ*Movac8* mutants were confirmed by DNA gel blot analysis and two independent mutants selected for further phenotypic analysis.

The *MoVAC8:GFP* construct was made by fusion PCR and standard restriction enzyme-mediated cloning. The *MoVAC8* gene (1.7 kbp promoter and 2.1 kbp CDS) was amplified with primers Vac8fusionFor and Vac8GFPRev. Primer Vac8GFPRev contained overhanging sequences at its 5′ end, which were complementary to the *sGFP* sequence. The 1.5 kb *sGFP* coding region, together with *TrpC* terminator, was amplified with primers GFPTrpCFor and GFPTrpCRev. The *MoVAC8:GFP* fusion cassette was then generated with primers Vac8fusionFor and GFPTrpCRev. An *XhoI* restriction enzyme recognition site was introduced into the 5′ end of both primers, Vac8fusionFor and GFPTrpCRev, to facilitate cloning of the *MoVAC8:GFP* fusion cassette into pCB1532 vector for fungal transformation [49]. The first 21 amino acid section of the N-terminus of MoVac8p, covering the predicted SH4 domain, was fused into the N-terminus of *GFP* and the yeast recombination method employed to generate a *MoVAC8SH4:GFP* fusion [50]. The 1.7 kb *MoVAC8* promoter sequence was amplified with primer pair, Vac8P-For and Vac8P-Rev, while SH4 domain-coding sequences were amplified with primer pair VAC8SH4-For and VAC8SH4-Rev from cDNA prepared from conidial total RNA. The amplicons were gel-purified and co-transformed into the relevant yeast strain together with plasmid pAGL1:GFP, which was linearized with *HindIII* and contains the selectable marker gene *SUR* conferring resistance to chlorimuron ethyl.

Site-directed mutageneses was performed on plasmid pCB1532-*MoVAC8:GFP* to generate alleles containing replacement of the predicted myristoylation (glycine) or palmitoylation (cysteine) modification sites by alanine residues, including *G2A*, *C4A*, *C8A*, *C9A*, *C4A/C8A*, *C4A/C9A*, *C8A/C9A* and *C4A/C8A/C9A*. In brief, a 714 bp region was amplified initially in two fragments, and nucleotide substitutions introduced into the primers, located at the overlapping region of the two fragments that were joined together by fusion PCR using primer pair Vac8mut-For and Vac8mut-Rev. The 714 bp fragment carrying the respective nucleotide substitutions was then digested with *FseI* and *PmlI* to release a 320 bp fragment that was used to replace the region spanning *FseI* and *PmlI* in plasmid pCB1532-*MoVAC8:GFP*. DNA sequencing was utilised to confirm successful introduction of each nucleotide substitution. Finally, the plasmid variants of pCB1532-*MoVAC8:GFP* were transformed into Δ*Movac8* mutants, and at least two independent transformants selected for phenotypic analysis. For complementation of Δ*Movac8* mutants, the pCB1532 vector carrying *MoVAC8:GFP* was transformed into a Δ*Movac8* mutant and at least two independent transformants tested for complementation.

### Targeted deletion of *MoTSC13*, *MoTSC13:GFP* fusion plasmid construction and Δ*Motsc13* complementation

Targeted gene deletion of *MoTSC13* was performed with the split-marker recombination method, as described above. The 1.0 kbp LF sequences were amplified with primers tsc1350.1 and tsc13m13f, while the 1.0 kbp RF sequences were amplified with primers tsc1330.1 and tsc13m13r. Generation of Δ*Motsc13* mutants was confirmed by DNA gel blot analysis and two independent mutants selected for further analysis [52]. The *MoTSC13:GFP* fusion was made using the yeast recombination method, as described above [53]. Briefly, the bialaphos resistance selectable marker gene BAR was amplified using primers BarF and BarR, and a 3.0 kb fragment of the *MoTSC13* gene amplified with primers Tsc13GFPFor and Tsc13GFPRev, *GFP-TrpC* terminator cassette amplified with primers GFPTrpCFor and GFPTrpR [52]. The amplicons were gel-purified and co-transformed into the relevant yeast strain together with vector pNEB-Nat which had been linearized with *Hind*III and *Sac*I.

### Expression of *MoVAC8* and *MoTSC13* in *S. cerevisiae*

Full-length double-stranded cDNAs of *MoVAC8* and *MoTSC13* were amplified from 1st strand cDNA using primer pair Vac8yeast50.1 and Vac8yeast30.1 and primer pair Tsc13yeast50.1 and Tsc13yeast30.1, respectively. *MoVAC8* cDNA was cloned into *KpnI* and *Xba*I sites of yeast expression vector pYES2 (Invitrogen) and introduced into *S. cerevisiae* strain BY4741 *vac8*Δ*::KANMX4*, while the *MoTSC13* cDNA was cloned between *Hind*III and *Xba*I sites of pYES2 and introduced into *S. cerevisiae* strain TDY2058. For expression of yeast-enhanced GFP (yEGFP) tagged *MoVAC8* and *MoTSC13* in *S. cerevisiae*, the cDNA of *MoVAC8* was amplified by primer pair Vac8yEGFPFor and Vac8yEGFPRev, *MoTSC13* amplified by Tsc13yEGFPFor and Tsc13yEGFPRev, and yEGFP amplified by primer pair yEGFPFor and yEGFPRev from plasmid pKT127 (obtained from EUROSCARF). The *MoVAC8:yEGFP* and *MoTSC13:yEGFP* fusion cassettes were generated by primer pair Vac8yEGFPFor and yEGFPRev, and primer pair Tsc13yEGFPFor and yEGFPRev respectively, both cloned between *EcoR*I and *Sph*I sites in pYES2 [53]. The *MoVAC8:yEGFP* construct was introduced into *S. cerevisiae* strain BY4741 *vac8Δ::KANMX4*, while *MoTSC13:yEGFP* was expressed into TDY2058. The cDNA sequences of both *MoVAC8* and *MoTSC13* in pYES2 were confirmed by DNA sequencing. All yeast transformants were confirmed by PCR and at least two independent yeast transformants chosen for analysis.Sensitivity to caffeine was assessed by spotting a dilution series of yeast cells (10^7^−10^4^ cells ml^−1^) on synthetic drop-out (SD) medium containing 0.05% or 0.1% caffeine in the presence of galactose. Vacuole morphology and inheritance in *S. cerevisiae* were observed by staining with FM4–64 (Molecular Probes, Invitrogen) according to [30]. For assessing vacuolar inheritance, pulse-chase labelling with FM4–64 [30] was performed by washing FM4–64 stained cells twice with fresh medium and incubating for an additional 4 h at 30°C. For testing temperature-sensitive lethality of yeast strains TDY2058, a dilution series of yeast cells (10^6^−10^4^ cells ml^−1^) expressing *MoTSC13* were spotted onto synthetic drop-out (SD) medium in the presence of galactose at 37°C, 30°C or 25°C.

### Generation of *M. oryzae* macroautophagy deficient strains carrying either *H1:RFP* (tdTomato), *MoVAC8:GFP* or *MoTSC13:GFP – gene fusions*

A *H1:RFP* fusion construct was introduced into Δ*Movac8* and Δ*Motsc13* mutants for live cell imaging of nuclei. *MoVAC8:GFP* and *MoTSC13:GFP* gene fusion constructs were introduced into Guy11 carrying *H1:RFP*. *H1:RFP*, *MoVAC8-GFP*. *MoTSC13:GFP* gene fusions were also introduced into a Δ*Moatg1* mutant to investigate behaviour of these fusion proteins in macroautophagy-deficient mutants. Transformants were selected by DNA gel blot, and at least two independent transformants investigated for all experiments. For targeted deletion of *MoATG4* in Guy11 expressing both *H1:RFP* and *MoVAC8:GFP* gene fusions, the split-marker *BAR* was used. Briefly, the two split *BAR* templates were amplified by primers M13F with BAsplit and M13R with ARsplit, and *MoATG4* LF amplified by primers Atg450.1 and Atg4m13f, *MoATG4* RF amplified by primers Atg430.1 and Atg4m13r, as previously described [11]. The LF-BA was obtained with primers Atg450.1 and BAsplit, while AR-RF obtained with primers ARsplit and Atg430.1.

### FM4–64 staining of conidia or mycelia in *M. oryzae*

The lipophilic styryl dye, FM4–64 (N-(3-triethylammoniumpropyl-)-4-(6(4-(diethylamino)phenyl) hexatrienyl) pyridinium dibromide) was used to stain vacuoles and endosomes of conidia or mycelia in *M. oryzae* (Molecular Probes, Invitrogen). Conidia grown in CM agar plate culture were collected with 4 ml of sterile distilled water, filtered through miracloth (Calbiochem). Approximately 200 µl of conidial suspension, at 1×10^6^ ml^−1^ was centrifuged at 6, 000 g for 5 min to precipitate conidia. After washing with 1 ml of sterile distilled water and centrifugation at 6, 000 g for 5 min, the conidial pellet was resuspended in 50 µl of liquid CM with 7.5 µM FM4–64. The suspension was incubated at 26°C for 20 min, and then conidia were recovered by centrifugation. The supernatant was discarded to remove excess FM4–64 and pellet washed twice with 1 ml of sterile distilled water. Conidia were finally resuspended in sterile distilled water at a concentration of 5×10^4^ ml^−1^. Appressorium development was observed on coverslips at indicated time points with epifluorescence microscopy.

### Plant pathogenicity and infection structure development assay

Cuticle penetration was assessed by recording the frequency of penetration peg formation from appressoria on onion epidermis. A 50 µl drop of conidial suspension at a concentration of 5×10^4^ conidia ml^−1^ was placed on the surface of onion epidermis and incubated in a humid environment at 24°C for 24 h or 48 h. The frequency of cuticle penetration was determined microscopically by counting formation of penetration pegs from at least 100 appressoria in triplicate replications of the experiment. Turgor generation in mature appressoria was measured by a cytorrhysis assay in a series of glycerol solutions of varying molarity, as previously described [6], [51]. Rice infections were performed using cultivar CO-39, a dwarf rice cultivar which is very susceptible to *M. oryzae* [46]. A conidial suspension (5×10^4^ mL^−1^) was produced by flooding 10-day-old *M. oryzae* culture plates with 0.2% (v/v) gelatine solution and the suspension spray-inoculated onto 14-day-old rice plants. Plants were placed in plastic bags for 24 h to maintain high humidity and then transferred to controlled environment chambers at 24°C and 90% relative humidity with illumination and 14 h light periods. Plants were incubated until disease symptoms were apparent 96–144 h later.

Conidial germination and development of appressoria were both monitored over time on hydrophobic borosilicate glass cover slips (Fisher Scientific) using a method adapted from [2], [46]. Conidial suspensions at 5×10^4^ conidia mL^−1^ were inoculated onto cover slips, incubated at 24°C, and all images of conidial germination and appressorium development were recorded using a Zeiss Axioskop 2 microscope (Zeiss).

### Light and epifluorescence microscopy

For epifluorescence microscopy of *GFP* or *RFP* expressing transformants, conidia were inoculated onto coverslips, incubated at 24°C and collected at indicated time points for observation using an IX81 motorized inverted microscope (Olympus) equipped with an UPlanSApo 100×/1.40 Oil objective (Olympus). Excitation of fluorescently-labeled proteins was carried out using a VS-LMS4 Laser-Merge-System with solid state lasers. The laser intensity was controlled by a VS-AOTF100 System and coupled into the light path using a VS-20 Laser-Lens-System (Visitron System). Images were captured using a Charged-Coupled Device camera (Photometric CoolSNAP HQ2, Roper Scientific). All parts of the system were under the control of the software package MetaMorph (Molecular Devices) and offline images were analyzed with MetaMorph software and Adobe Photoshop CS2 (Adobe Systems Incorporated).

## Supporting Information

Figure S1 **ClustalW alignment of Vac8p and Tsc13p between** ***M.oryzae*** **and** ***S. cerevisiae*** **.** (**A**) Vac8p ClustalW alignment. (**B**) Tsc13p ClustalW alignment. Star indicates conserved amino acids shown to be important for function of Tsc13p of *S. cerevisiae*.
(TIFF)

Figure S2 **Targeted deletion of** ***MoVAC8*** **,** ***MoTSC13*** **and** ***MoATG4*** **genes in** ***M. oryzae*** **.** A) Southern blot analysis was used to confirm targeted deletion in Δ*Movac8* mutants. *MoVAC8* left flanking region, *MoVAC8* ORF, and Hygromycin resistance marker gene fragment HY were used as probes. Δ*Movac8.*3, 10, 11, 18 and 21 were defined as five independent deletion mutants, and strains 8.6 and *8.*15 were detected as ectopic insertion mutants. Two independent deletion mutants Δ*Movac8.*10 and Δ*Movac8.*21 were chosen for further phenotypic analysis. (**B**) Southern blot analysis was used to confirm targeted deletion in Δ*Motsc13* mutants. *MoTSC13* left flanking region, *MoTsc13* ORF, and Hygromycin resistance marker gene fragment HY were used as probes. Δ*Motsc13.*4 and Δ*Motsc13*.8 were two independent knockout mutants, and Δ*Motsc13.*1 and Δ*Motsc13*.5 were ectopic insertion mutants. Δ*Motsc13.*2 and Δ*Motsc13.*3 were Δ*Motsc13* mutants in the Δ*ku70* background strain [11]. Two independent deletion mutants, Δ*Motsc13.*4 and Δ*Motsc13.*8, were chosen for further analysis. (**C**) Southern blot analysis was used to confirm putative Δ*Moatg4* mutants. *MoATG4* left flanking region, ORF, and *BAR* marker gene were used as probes. Δ*Moatg4.*5, 8, 10, 14 and 16 and 18 were defined as six independent deletion mutants, and strains 13, 15 and 19 were detected as ectopic insertion mutants. Two independent deletion mutant Δ*Moatg4.8* and Δ*Moatg4.18* were chosen for further analysis.
(TIFF)

Figure S3 **Myristoylation and palmitoylation of MoVac8p are required for association of MoVac8p with vacuolar membranes in vegetative hyphae.** (**A**) N-terminal sequences of MoVAC8p-GFP variants used in this study. Alanine mutations within the N-terminal SH4 domain are indicated in bold. Constructs were named according to their mutated glycine or cysteine residues and numbers indicate the amino acid positions within the SH4 domain. (**B**) Localization of MoVac8p-GFP variant proteins in vegetative hyphae. Δ*Movac8* mutant was transformed with constructs expressing the indicated GFP fusion proteins. Vegetative hyphae of transformants expressing MoVac8p-GFP variant fusion proteins were prepared and visualised by epifluorescence microscopy, as indicated in Figure 7. Arrows indicate the position of mis-localised MoVac8p-GFP in the septal pore area. (**C**) Effects of SH4 domain mutations in localisation of MoVac8p-GFP. MoVac8p-GFP fusion proteins were associated with the vacuolar membrane, while mutations within the SH4 domain resulted in mislocalisation of fusion proteins in the septal pore area. Vegetative hyphae expressing each respective MoVac8p-GFP allele were grown in CM for 24 h, followed by CFW staining of cell wall and septa before fluorescence microscopy. The number in parentheses indicates the total number of septa counted and examined by CFW staining in epifluorescence microscopy experiments, and the numbers above the grey bars represent the percentage of septa enriched with mislocalised MoVac8p-GFP fusion proteins. Scale bar = 10 µm.
(TIFF)

Figure S4 **Palmitoylation of MoVac8p is required for association of Vac8p with the vacuolar membrane in conidia and appressoria.** Conidia of Δ*Movac8* mutants expressing the variant MoVac8p-GFP fusion proteins were stained with FM4–64, as described above and localisation of the respective GFP fusion proteins in conidia and appressoria analysed by epifluorescence microscopy. Arrows indicate the position of mis-localised MoVac8p-GFP in the septal pore area. Scale bar = 10 µm.
(TIFF)

Figure S5 **Functional analysis of MoVac8p myristoylation and palmitoylation mutants in caffeine resistance.** The *MoVAC8:GFP* fusion construct and each mutant allele were transformed into the Δ*Movac8* mutant, and three independent transformants for each construct grown on CM plates in the presence of 0.1% caffeine for 15 days.
(TIF)

Figure S6 **Macroautophagy core gene** ***MoATG1*** **is necessary for vacuole degeneration and ER degeneration during appressorium development in** ***M. oryzae*** **.** Left Panels. Degradation of perinuclear and peripheral ER membrane-associated protein, MoTsc13p-GFP, was blocked in Δ*Moatg1* mutants during appressorium development. Right Panels. Degradation of vacuolar membrane protein MoVac8p-GFP was blocked in Δ*Moatg1* mutants during appressorium development. Scale bar = 10 µm.
(TIFF)

Figure S7 ***MoVAC8*** **is not required for vegetative growth on different stress medium except caffeine.** Uniformly sized mycelial plugs were used to inoculate agar plate cultures supplemented with Congo Red, Calcofluor white (CFW), Sodium dodecyl sulfate (SDS), hydrogen peroxide or caffeine, as shown, and incubated for 12 days at 24°C.
(TIF)

Figure S8 ***MoTSC13*** **is involved in maintaining cell wall integrity and hyper osmotic stress adaptation, but not appressorium development or turgor generation.** (**A**) Disruption of *MoTSC13* reduced hyphal growth of *M. oryzae* on CM, and made *M. oryzae* sensitive to hyper osmotic stress and cell wall stress. (**B**) Vegetative growth was impaired by deletion of *MoTSC13*. The diameter of colonies of both Guy11 and Δ*Motsc13* mutants grown on CM plates was recorded at indicated times in the line graph presented. (**C**) Conidiation was reduced by deletion of *MoTSC13*. A 3 mm mycelium plug was inoculated in triplicate and incubated at 24°C for 12 days. Conidia generated were collected in 3 ml of distilled water, and 20 µl of conidial suspension used for counting on a hemacytometer. Bar chart shows conidia per cm2 of plate cultures. (**D**) *MoTSC13* is dispensable for appressorium development. Bar charts showing the percentage of conidia formaing an appressorium after 6 h or 24 h (**E**) *MoTSC13* is dispensable for turgor generation in the appressorium. Bar charts showing the percentage of cell collapse upon incubation in increasing concentrations of glycerol [6].
(TIFF)

Table S1 Detailed information of oligonucleotide primers used in this study.
(DOCX)
