# Supplementary figures and images for: Infection-Associated Nuclear Degeneration in the Rice Blast Fungus Magnaporthe oryzae Requires Non-Selective Macro-Autophagy
Source: PLoS One. 2012 Mar 20;7(3):e33270. doi: 10.1371/journal.pone.0033270 (PMC3308974; doi:10.1371/journal.pone.0033270)

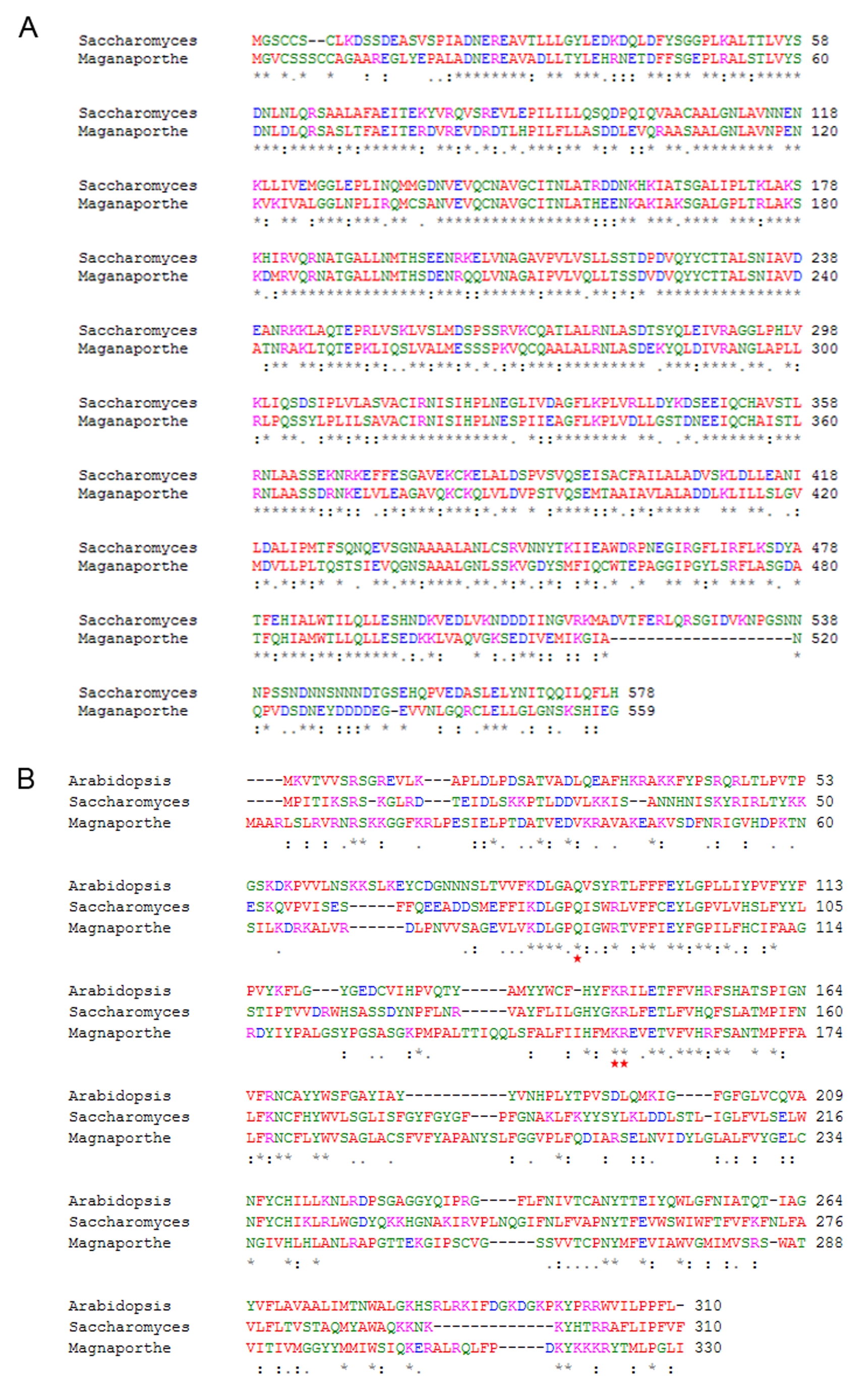

Supplement: Figure S1 — ClustalW alignment of Vac8p and Tsc13p between M.oryzae and S. cerevisiae . (A) Vac8p ClustalW alignment. (B) Tsc13p ClustalW alignment. Star indicates conserved amino acids shown to be important for function of Tsc13p of S. cerevisiae. (TIFF) [file pone.0033270.s001.tif]

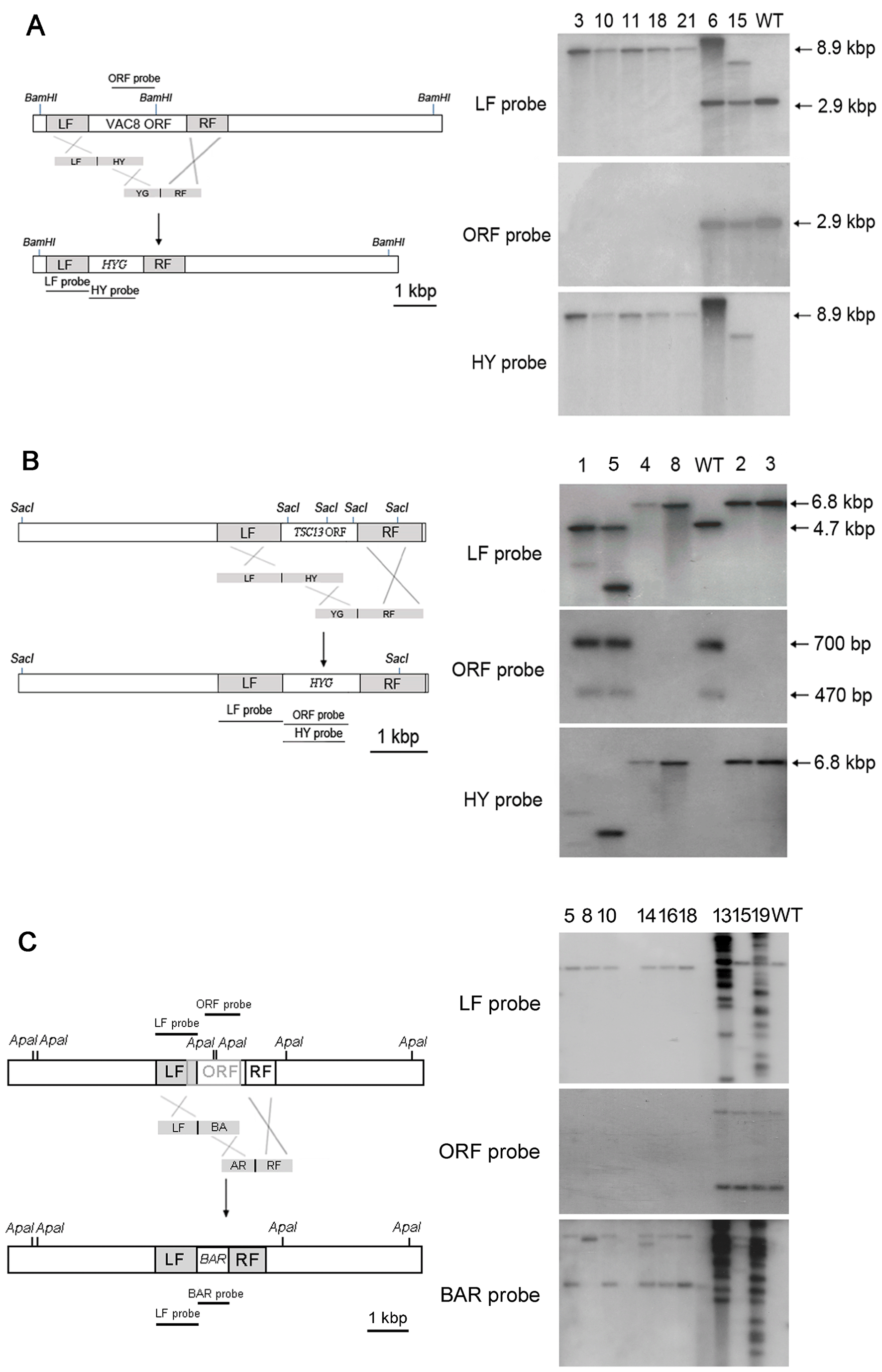

Supplement: Figure S2 — Targeted deletion of MoVAC8 , MoTSC13 and MoATG4 genes in M. oryzae . A) Southern blot analysis was used to confirm targeted deletion in ΔMovac8 mutants. MoVAC8 left flanking region, MoVAC8 ORF, and Hygromycin resistance marker gene fragment HY were used as probes. ΔMovac8.3, 10, 11, 18 and 21 were defined as five independent deletion mutants, and strains 8.6 and 8.15 were detected as ectopic insertion mutants. Two independent deletion mutants ΔMovac8.10 and ΔMovac8.21 were chosen for further phenotypic analysis. (B) Southern blot analysis was used to confirm targeted deletion in ΔMotsc13 mutants. MoTSC13 left flanking region, MoTsc13 ORF, and Hygromycin resistance marker gene fragment HY were used as probes. ΔMotsc13.4 and ΔMotsc13.8 were two independent knockout mutants, and ΔMotsc13.1 and ΔMotsc13.5 were ectopic insertion mutants. ΔMotsc13.2 and ΔMotsc13.3 were ΔMotsc13 mutants in the Δku70 background strain [11]. Two independent deletion mutants, ΔMotsc13.4 and ΔMotsc13.8, were chosen for further analysis. (C) Southern blot analysis was used to confirm putative ΔMoatg4 mutants. MoATG4 left flanking region, ORF, and BAR marker gene were used as probes. ΔMoatg4.5, 8, 10, 14 and 16 and 18 were defined as six independent deletion mutants, and strains 13, 15 and 19 were detected as ectopic insertion mutants. Two independent deletion mutant ΔMoatg4.8 and ΔMoatg4.18 were chosen for further analysis. (TIFF) [file pone.0033270.s002.tif]

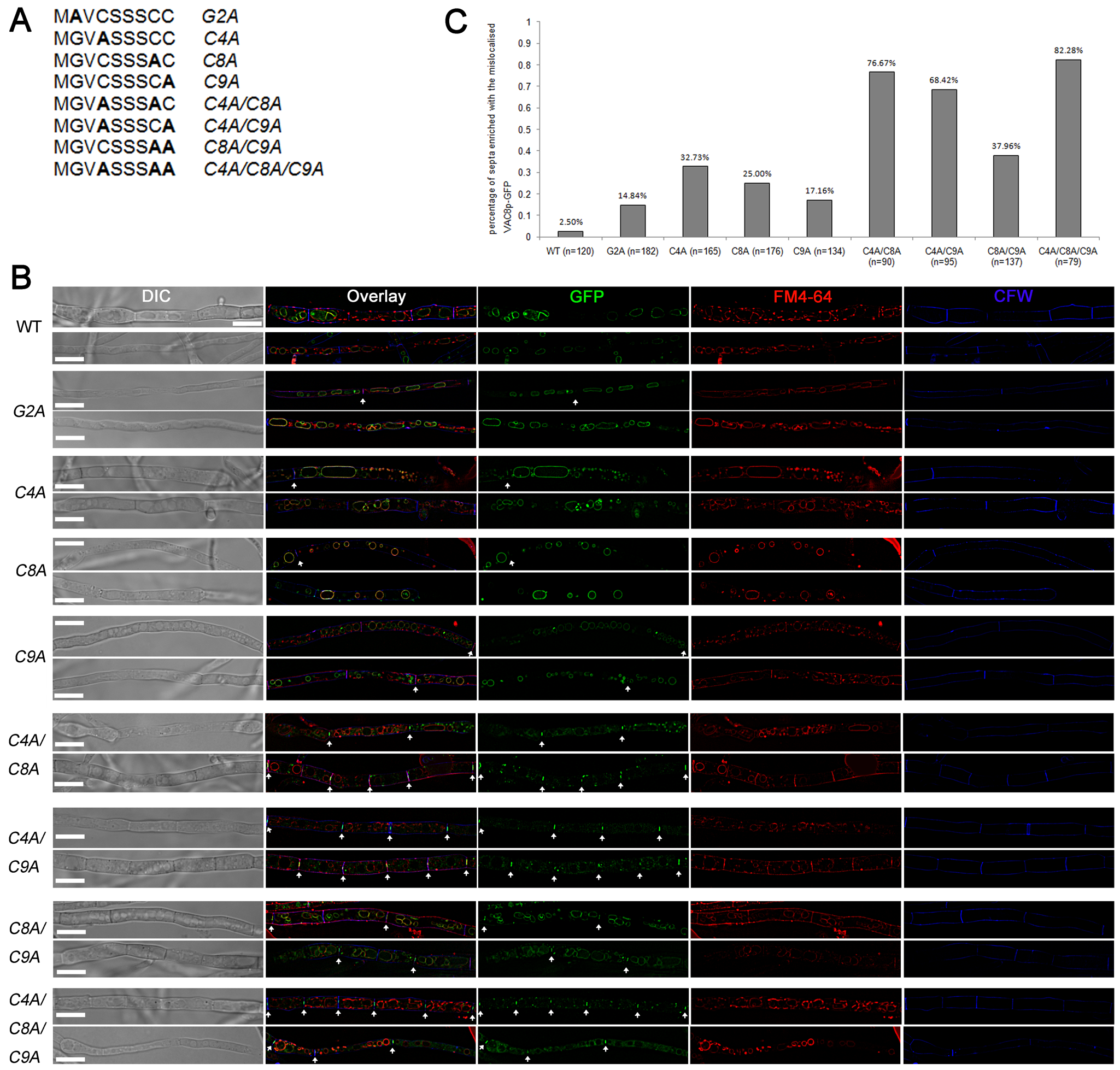

Supplement: Figure S3 — Myristoylation and palmitoylation of MoVac8p are required for association of MoVac8p with vacuolar membranes in vegetative hyphae. (A) N-terminal sequences of MoVAC8p-GFP variants used in this study. Alanine mutations within the N-terminal SH4 domain are indicated in bold. Constructs were named according to their mutated glycine or cysteine residues and numbers indicate the amino acid positions within the SH4 domain. (B) Localization of MoVac8p-GFP variant proteins in vegetative hyphae. ΔMovac8 mutant was transformed with constructs expressing the indicated GFP fusion proteins. Vegetative hyphae of transformants expressing MoVac8p-GFP variant fusion proteins were prepared and visualised by epifluorescence microscopy, as indicated in Figure 7. Arrows indicate the position of mis-localised MoVac8p-GFP in the septal pore area. (C) Effects of SH4 domain mutations in localisation of MoVac8p-GFP. MoVac8p-GFP fusion proteins were associated with the vacuolar membrane, while mutations within the SH4 domain resulted in mislocalisation of fusion proteins in the septal pore area. Vegetative hyphae expressing each respective MoVac8p-GFP allele were grown in CM for 24 h, followed by CFW staining of cell wall and septa before fluorescence microscopy. The number in parentheses indicates the total number of septa counted and examined by CFW staining in epifluorescence microscopy experiments, and the numbers above the grey bars represent the percentage of septa enriched with mislocalised MoVac8p-GFP fusion proteins. Scale bar = 10 µm. (TIFF) [file pone.0033270.s003.tif]

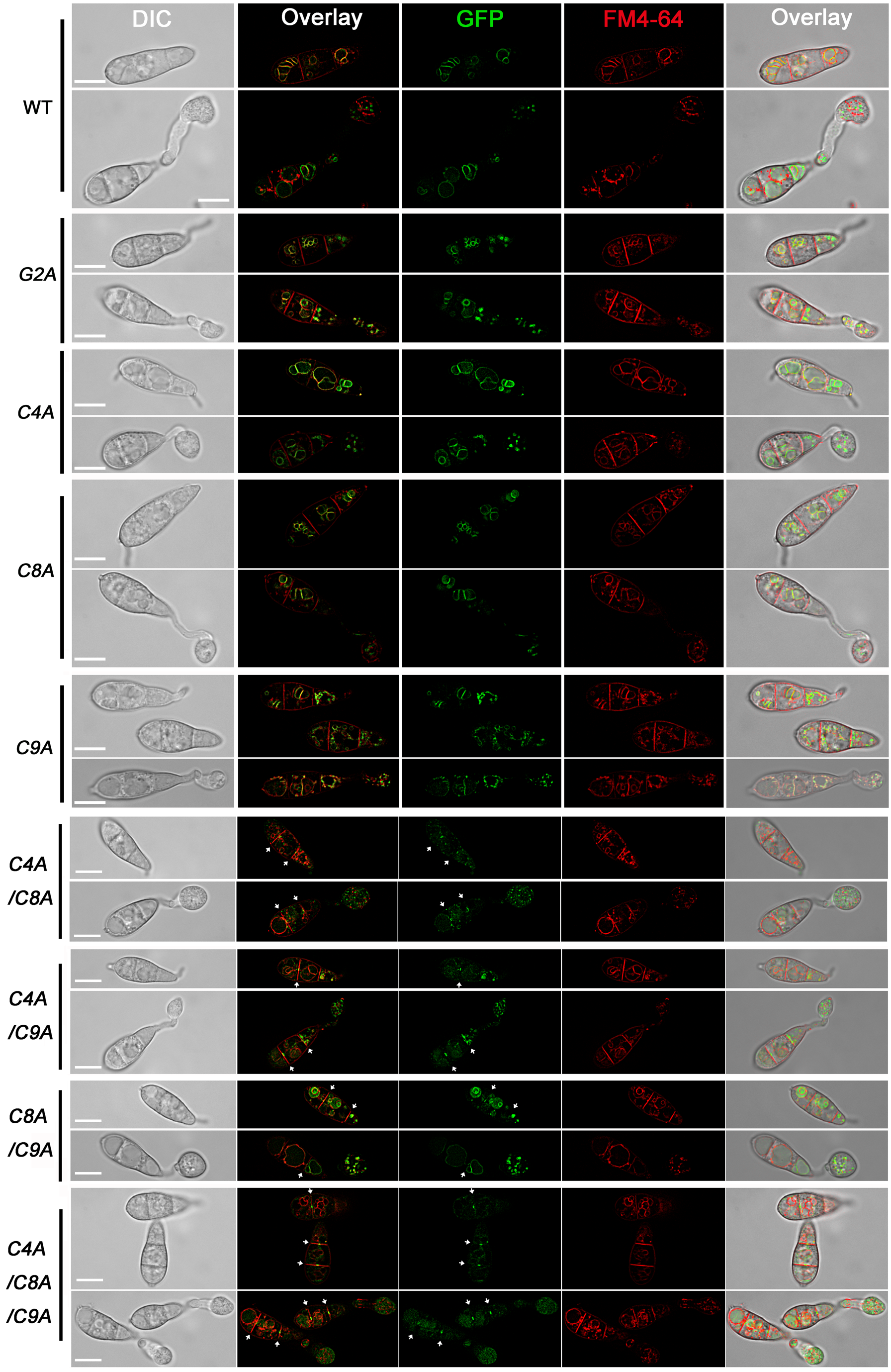

Supplement: Figure S4 — Palmitoylation of MoVac8p is required for association of Vac8p with the vacuolar membrane in conidia and appressoria. Conidia of ΔMovac8 mutants expressing the variant MoVac8p-GFP fusion proteins were stained with FM4–64, as described above and localisation of the respective GFP fusion proteins in conidia and appressoria analysed by epifluorescence microscopy. Arrows indicate the position of mis-localised MoVac8p-GFP in the septal pore area. Scale bar = 10 µm. (TIFF) [file pone.0033270.s004.tif]

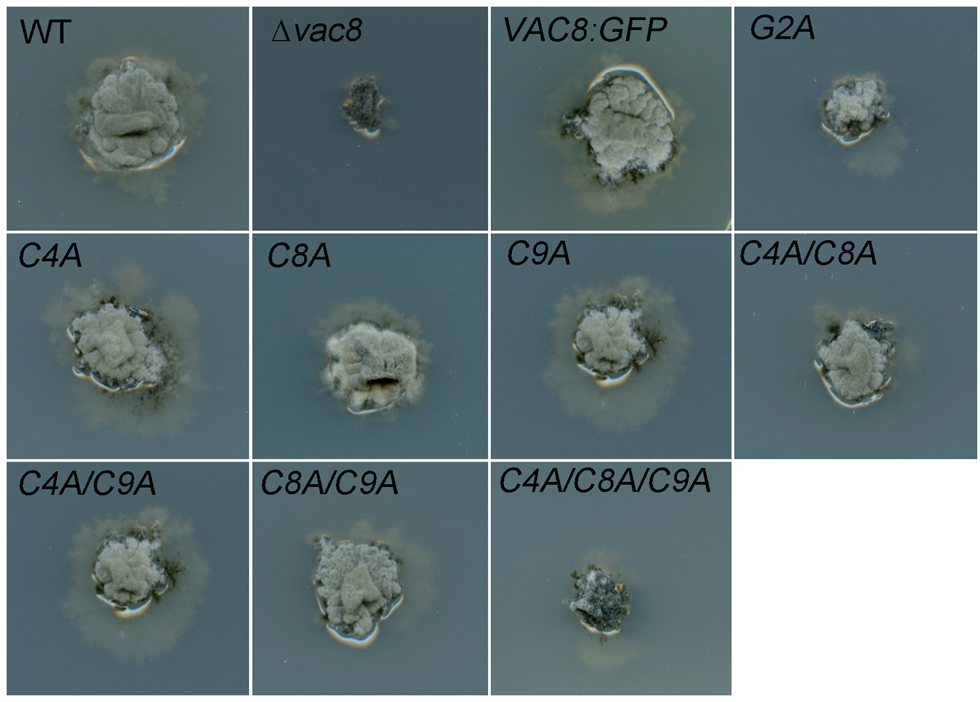

Supplement: Figure S5 — Functional analysis of MoVac8p myristoylation and palmitoylation mutants in caffeine resistance. The MoVAC8:GFP fusion construct and each mutant allele were transformed into the ΔMovac8 mutant, and three independent transformants for each construct grown on CM plates in the presence of 0.1% caffeine for 15 days. (TIF) [file pone.0033270.s005.tif]

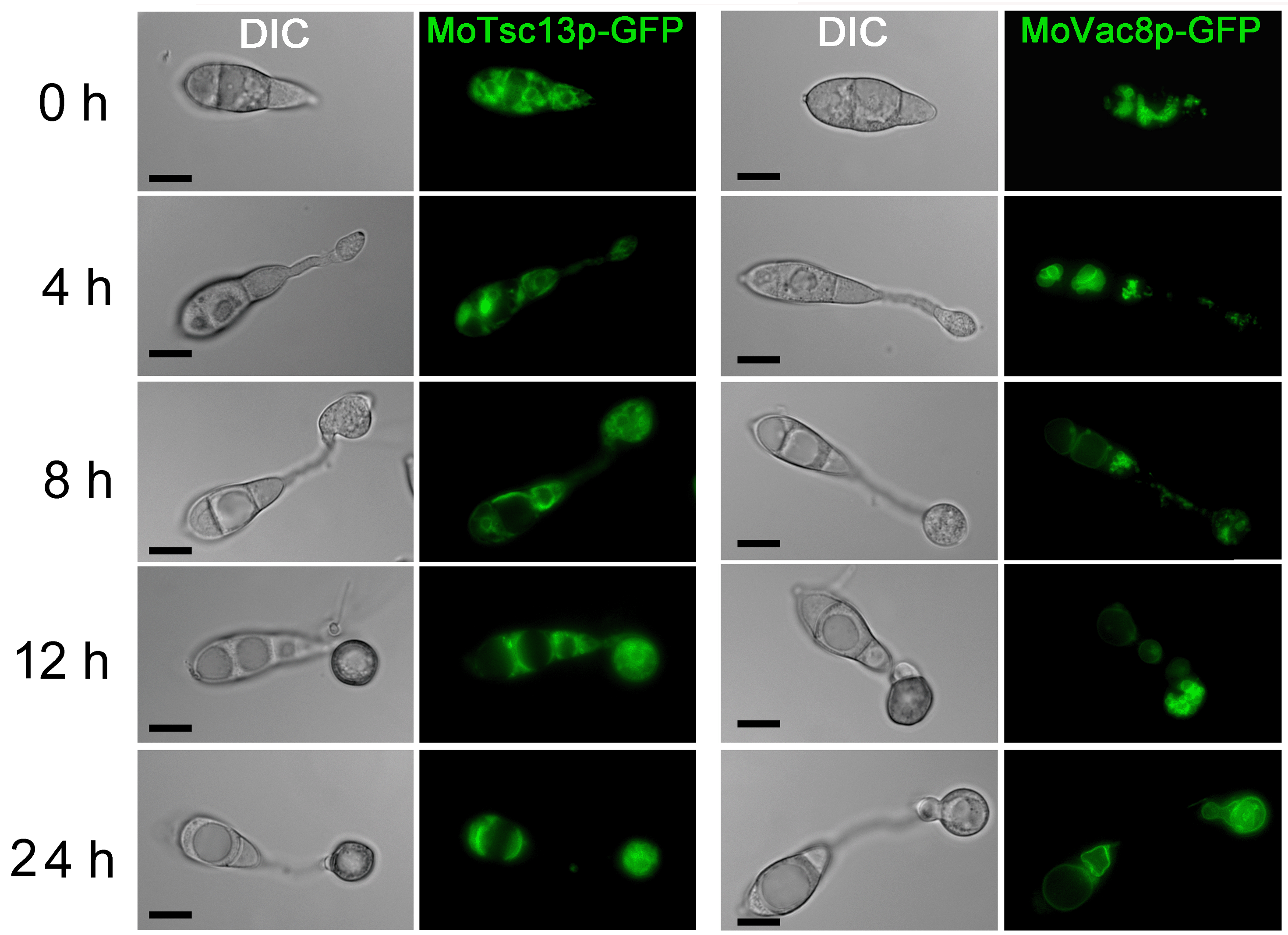

Supplement: Figure S6 — Macroautophagy core gene MoATG1 is necessary for vacuole degeneration and ER degeneration during appressorium development in M. oryzae . Left Panels. Degradation of perinuclear and peripheral ER membrane-associated protein, MoTsc13p-GFP, was blocked in ΔMoatg1 mutants during appressorium development. Right Panels. Degradation of vacuolar membrane protein MoVac8p-GFP was blocked in ΔMoatg1 mutants during appressorium development. Scale bar = 10 µm. (TIFF) [file pone.0033270.s006.tif]

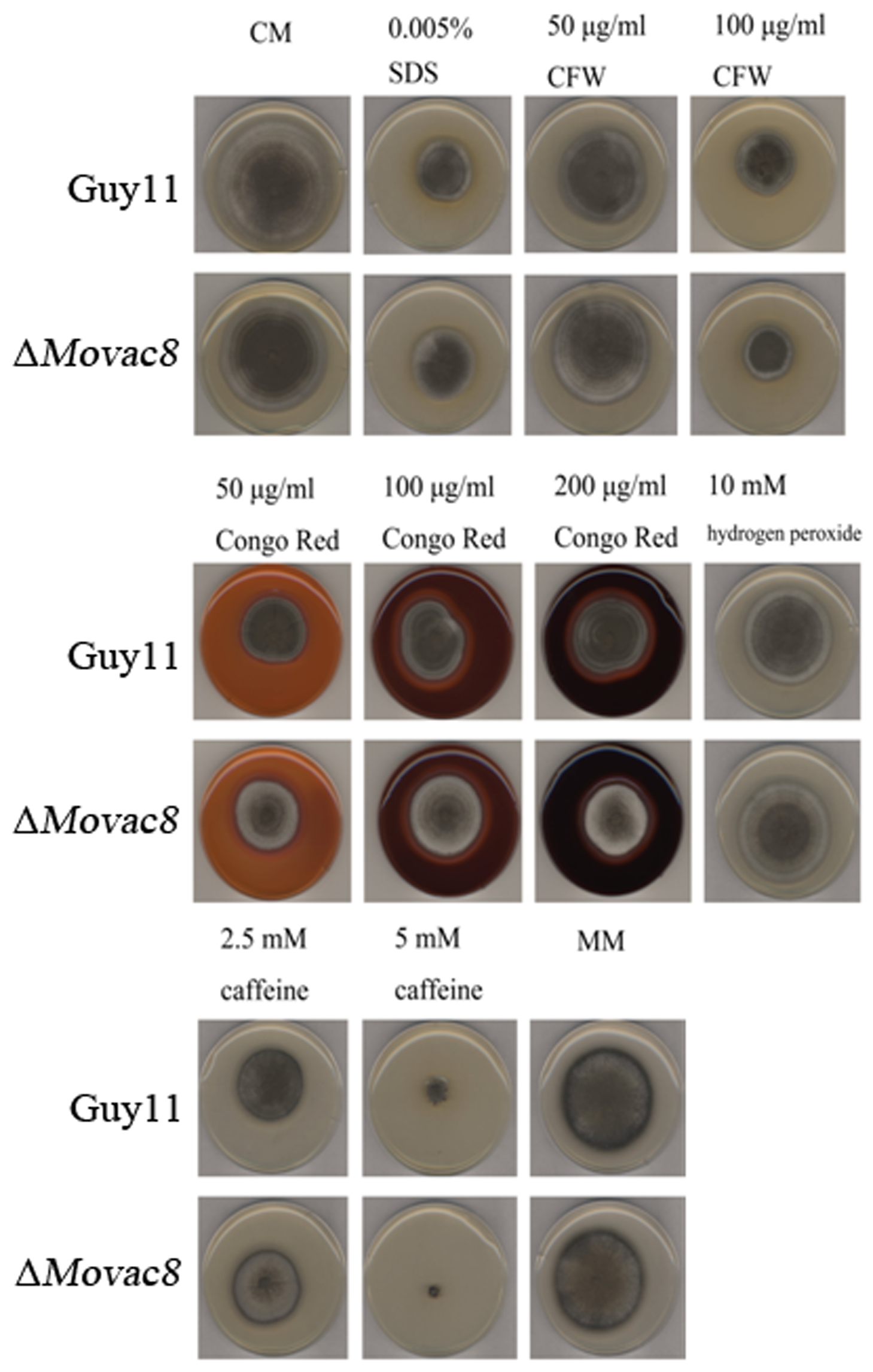

Supplement: Figure S7 — MoVAC8 is not required for vegetative growth on different stress medium except caffeine. Uniformly sized mycelial plugs were used to inoculate agar plate cultures supplemented with Congo Red, Calcofluor white (CFW), Sodium dodecyl sulfate (SDS), hydrogen peroxide or caffeine, as shown, and incubated for 12 days at 24°C. (TIF) [file pone.0033270.s007.tif]

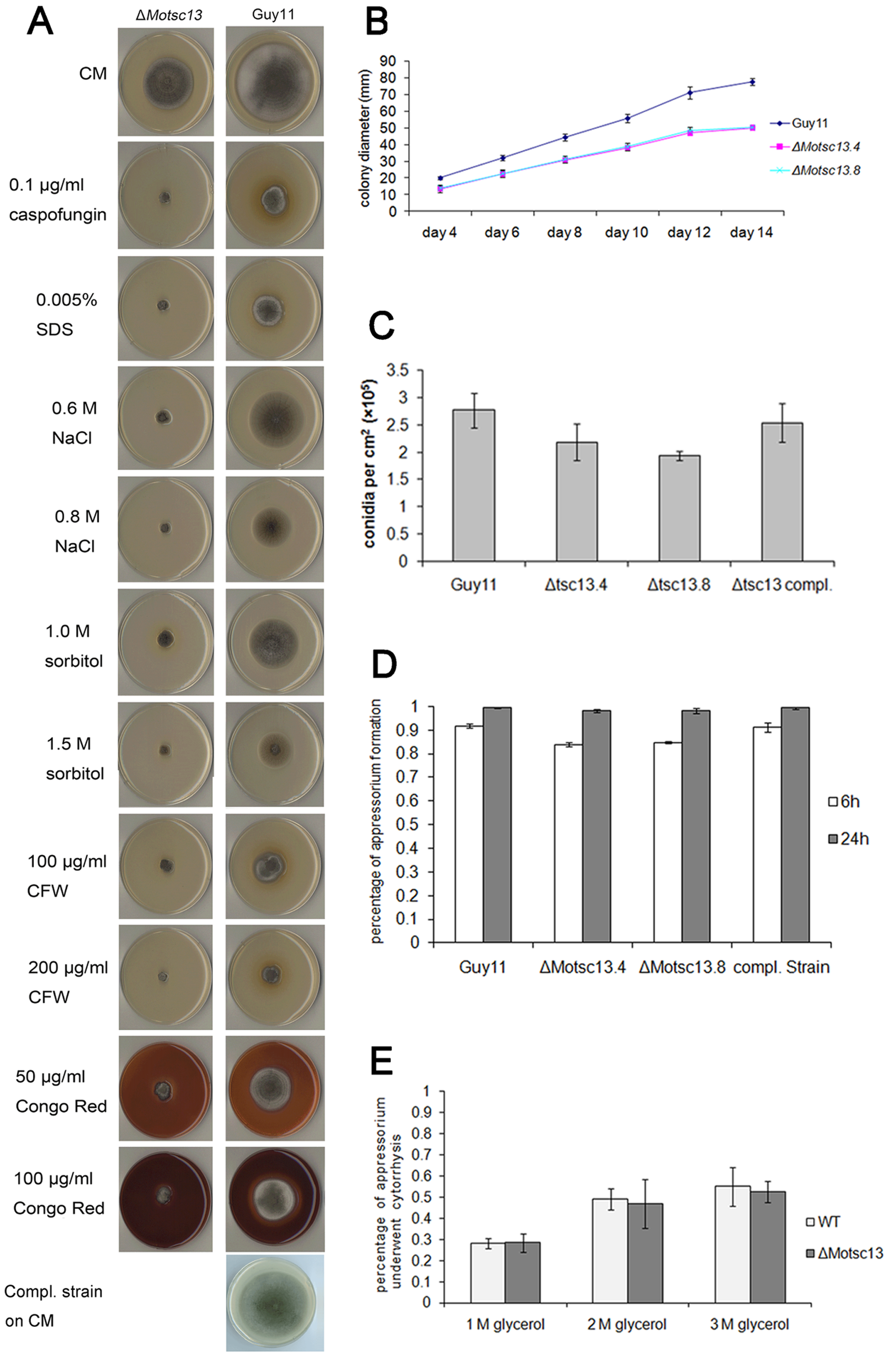

Supplement: Figure S8 — MoTSC13 is involved in maintaining cell wall integrity and hyper osmotic stress adaptation, but not appressorium development or turgor generation. (A) Disruption of MoTSC13 reduced hyphal growth of M. oryzae on CM, and made M. oryzae sensitive to hyper osmotic stress and cell wall stress. (B) Vegetative growth was impaired by deletion of MoTSC13. The diameter of colonies of both Guy11 and ΔMotsc13 mutants grown on CM plates was recorded at indicated times in the line graph presented. (C) Conidiation was reduced by deletion of MoTSC13. A 3 mm mycelium plug was inoculated in triplicate and incubated at 24°C for 12 days. Conidia generated were collected in 3 ml of distilled water, and 20 µl of conidial suspension used for counting on a hemacytometer. Bar chart shows conidia per cm2 of plate cultures. (D) MoTSC13 is dispensable for appressorium development. Bar charts showing the percentage of conidia formaing an appressorium after 6 h or 24 h (E) MoTSC13 is dispensable for turgor generation in the appressorium. Bar charts showing the percentage of cell collapse upon incubation in increasing concentrations of glycerol [6]. (TIFF) [file pone.0033270.s008.tif]
