# Supplementary material for: Infection-Associated Nuclear Degeneration in the Rice Blast Fungus Magnaporthe oryzae Requires Non-Selective Macro-Autophagy
Source: PLoS One. 2012 Mar 20;7(3):e33270. doi: 10.1371/journal.pone.0033270 (PMC3308974; doi:10.1371/journal.pone.0033270)
Supplement: Table S1 — Detailed information of oligonucleotide primers used in this study. (DOCX) [file pone.0033270.s009.docx]

**Table S1.** Detailed information of primers used in this study.

| Primer name | Primer sequence (5' -3') | Strand | Template | Amplicon |
| --- | --- | --- | --- | --- |
| M13F | cgccagggttttcccagtcacgac | + | pCB1004 [49] | Split HY of selection marker gene *HYG* for targeted gene deletion |
| HYsplit | ggatgcctccgctcgaagta | - | pCB1004 [49] | Split HY of selection marker gene *HYG* for targeted gene deletion |
| YGsplit | cgttgcaagacctgcctgaa | + | pCB1004 [49] | Split YG of selection marker gene *HYG* for targeted gene deletion |
| M13R | agcggataacaatttcacacagga | - | pCB1004 [49] | Split YG of selection marker gene *HYG* for targeted gene deletion |
| vac850.1 | GGTCCGCTTTCAGGCTTTCCCAGA | + | *M. oryzae* Guy-  11 genomic  DNA | *MoVAC8* RF for targeted gene deletion |
| vac8m13f | GTCGTGACTGGGAAAACCCTGGCGCTGCCCCCAATCCAAGAGCGTGTT | - | *M. oryzae* Guy-  11 genomic  DNA | *MoVAC8* RF for targeted gene deletion |
| vac830.1 | CTCCTTCTTTACCTCGGCCTTTAG | - | *M. oryzae* Guy-  11 genomic  DNA | *MoVAC8* LF for targeted gene deletion |
| vac8m13r | TCCTGTGTGAAATTGTTATCCGCTGTTCAGGACCACCAATCATACCGG | + | *M. oryzae* Guy-  11 genomic  DNA | *MoVAC8* LF for targeted gene deletion |
| Vac8fusionFor | CCG*CTCGAG*CAGGCACATCATCCATCA | + | *M. oryzae* Guy-  11 genomic  DNA | *MoVAC8* promoter and CDS for *MoVAC8:GFP* fusion construct |
| Vac8GFPRev | CAGCTCCTCGCCCTTGCTCACCATGCCTTCAATGTGTGATTTCG | - | *M. oryzae* Guy-  11 genomic  DNA | *MoVAC8* promoter and CDS for *MoVAC8:GFP* fusion construct |
| GFPTrpCFor | ATGGTGAGCAAGGGCGAGGAGCTG | + | pMJK-80  [52] | GFP CDS and terminator for *MoVAC8:GFP* construct |
| GFPTrpCRev | CCG*CTCGAG*GTGGAGATGTGGAGTGGGCGC | - | pMJK-80  [52] | GFP CDS and terminator for *MoVAC8:GFP* construct |
| Vac8P-For | GATAATGGGAATTGATTATTGCACGGGAATCTCGAGCAGGCACATCATCCATCA | + | *M. oryzae* Guy-  11 genomic  DNA | *MoVAC8* promoter for *MoVAC8SH4:GFP* fusion construct |
|  |  |  |  |  |
| Vac8P-Rev | TTTCCGTTTTTCTATATTAGGTAC | - | *M. oryzae* Guy-  11 genomic  DNA | *MoVAC8* promoter for *MoVAC8SH4:GFP* fusion construct |
| Vac8SH4-For | TCATTCGTACCTAATATAGAAAAACGGAAAATGGGTGTTTGCAGCTCATCATGC | + | 1^st^ cDNA reverse-transcried from *M. oryzae* Guy-  11 conidial total RNA | *MoVAC8* SH4 domain for *MoVAC8SH4:GFP* fusion construct |
| Vac8SH4-Rev | GGTGAACAGCTCCTCGCCCTTGCTCACCATTGCAGGCTCGTACAAACCC | - | 1^st^ cDNA reverse-transcried from *M. oryzae* Guy-  11 conidial total RNA | *MoVAC8* SH4 domain for *MoVAC8SH4:GFP* fusion construct |
| Vac8mut-For | ATGCAATCAGTGAACAAGGTCAT | + |  | The 714 bp fragments containing region spanning *FseI* and *PmlI* for the site-directed mutageneses of pCB1532-*MoVAC8:GFP* |
|  |  |  |  |  |
| Vac8mut-Rev | TTATGCGCCATCCAATCTTTCG | - |  | The 714 bp fragments containing region spanning *FseI* and *PmlI* for the site-directed mutageneses of pCB1532-*MoVAC8:GFP* |
| Vac8G2A | GAAAAACGGAAAATG*GCC*GTTTGCAGCTCATCATGC | + | pCB1532-*MoVAC8:GFP* | Together with primer Vac8mut-Rev to amplify one fragment carrying G2A |
| Vac8G2A-Rev | GAGCTGCAAAC*GGC*CATTTTCCGTTTTTC | - | pCB1532-*MoVAC8:GFP* | Together with primer Vac8mut-For to amplify the other fragment carrying G2A |
| Vac8C4A | CGGAAAATGGGTGTT*GCC*AGCTCATCATGCTGCGC | + | pCB1532-*MoVAC8:GFP* | Together with primer Vac8mut-Rev to amplify one fragment carrying C4A |
| Vac8C4A-Rev | CATGATGAGCT*GGC*AACACCCATTTTCCG | - | pCB1532-*MoVAC8:GFP* | Together with primer Vac8mut-For to amplify the other fragment carrying C4A |
| Vac8C8A | GGTGTTTGCAGCTCATCA*GCC*TGCGCAGGTACGGCTTTTC | + | pCB1532-*MoVAC8:GFP* | Together with primer Vac8mut-Rev to amplify one fragment carrying C8A |
| Vac8C8A-Rev | CCTGCGCA*GGC*TGATGAGCTGCAAACACC | - | pCB1532-*MoVAC8:GFP* | Together with primer Vac8mut-For to amplify the other fragment carrying C8A |
| Vac8C9A | GTTTGCAGCTCATCATGC*GCC*GCAGGTACGGCTTTTC | + | pCB1532-*MoVAC8:GFP* | Together with primer Vac8mut-Rev to amplify one fragment carrying C9A |
| Vac8C9A-Rev | GTACCTGC*GGC*GCATGATGAGCTGCAAAC | - | pCB1532-*MoVAC8:GFP* | Together with primer Vac8mut-For to amplify one fragment carrying C9A |
| Vac8C489A | GAAAAACGGAAAATGGGTGTT*GCC*AGCTCATCA*GCCGCC*GCAGGTACGGCTTTTCTACCGC | + | pCB1532-*MoVAC8:GFP* | Together with primer Vac8mut-Rev to amplify one fragment carrying C4A/C8A/C9A |
| Vac8C489A-Rev | GTACCTGC*GGCGGC*TGATGAGCT*GGC*AACACCCATTTTCCGTTTTTC | - | pCB1532-*MoVAC8:GFP* | Together with primer Vac8mut-For to amplify the other fragment carrying C4A/C8A/C9A |
| Vac8C48A | GAAAAACGGAAAATGGGTGTT*GCC*AGCTCATCA*GCC*TGCGCAGGTACGGCTTTTCTACCGC | + | pCB1532-*MoVAC8:GFP* | Together with primer Vac8mut-Rev to amplify one fragment carrying C4A/C8A |
| Vac8C48A-Rev | CTGCGCA*GGC*TGATGAGCT*GGC*AACACCCATTTTCCGTTTTTC | - | pCB1532-*MoVAC8:GFP* | Together with primer Vac8mut-For to amplify the other fragment carrying C4A/C8A |
| Vac8C49A | GAAAAACGGAAAATGGGTGTT*GCC*AGCTCATCATGC*GCC*GCAGGTACGGCTTTTCTACCGC | + | pCB1532-*MoVAC8:GFP* | Together with primer Vac8mut-Rev to amplify one fragment carrying C4A /C9A |
| Vac8C49A-Rev | C*GGC*GCATGATGAGCT*GGC*AACACCCATTTTCCGTTTTTC | - | pCB1532-*MoVAC8:GFP* | Together with primer Vac8mut-For to amplify the other fragment carrying C4A /C9A |
| Vac8C89A | GGGTGTTTGCAGCTCATCA*GCCGCC*GCAGGTACGGCTTTTCTACCGC | + | pCB1532-*MoVAC8:GFP* | Together with primer Vac8mut-Rev to amplify one fragment carrying C8A/C9A |
| Vac8C89A-Rev | CCTGC*GGCGGC*TGATGAGCTGCAAACACCC | - | pCB1532-*MoVAC8:GFP* | Together with primer Vac8mut-For to amplify the other fragment carrying C8A/C9A |
| tsc1350.1 | TCATGTCAGAACCCTCCTCCAAAA | + | *M. oryzae* Guy-  11 genomic  DNA | MoTSC13 RF for targeted gene deletion |
| tsc13m13f | GTCGTGACTGGGAAAACCCTGGCGTGATTATTGGTTGGTGGTATGTAG | - | *M. oryzae* Guy-  11 genomic  DNA | *MoTSC13* RF for targeted gene deletion |
| tsc1330.1 | ATCGCCTGAATCACATCAAACA | - | *M. oryzae* Guy-  11 genomic  DNA | *MoTSC13* LF for targeted gene deletion |
| tsc13m13r | TCCTGTGTGAAATTGTTATCCGCTGCACGGTGACAAGCAAAGAGTT | + | *M. oryzae* Guy-  11 genomic  DNA | *MoTSC13* LF for targeted gene deletion |
| BarF | AACTGTTGGGAAGGGCGATCGGTGCGGGCCGTCGACAGAAGATGATATTGAAGG | + | pCB1530 [49] | Selection marker gene *BAR* for *MoTSC13:GFP* construct |
| BarR | GTCGACCTAAATCTCGGTGACGGG | - | pCB1530 [49] | Selection marker gene *BAR* for *MoTSC13:GFP* construct |
| Tsc13GFPFor | GTCCTGCCCGTCACCGAGATTTAGGTCGACTTACGATTTTCACCTACCTCCC | + | *M. oryzae* Guy-  11 genomic  DNA | *MoTSC13* promoter and CDS for *MoTSC13:GFP* construct |
| Tsc13GFPRev | GGTGAACAGCTCCTCGCCCTTGCTCACCATAATGAGACCAGGAAGCATAGTG | - | *M. oryzae* Guy-  11 genomic  DNA | *MoTSC13* promoter and CDS for *MoTSC13:GFP* construct |
| GFPTrpCFor | ATGGTGAGCAAGGGCGAGGAGCTG | + | pMJK-80  [52] | GFP CDS and terminator for *MoTSC13:GFP* construct |
| GFPTrpR | TTCACACAGGAAACAGCTATGACCATGATTGTGGAGATGTGGAGTGGGCGC | - | pMJK-80  [52] | GFP CDS and terminator for *MoTSC13:GFP* construct |
| oligo(dT) anchoring primer | AAGCAGTGGTATCAACGCAGAGTAC(T)_30_VN |  | *M. oryzae* Guy-  11 conidial total RNA | 1^st^ cDNA from total RNA |
| Vac8yeast50.1 | *GGTACC*ATGGGTGTTTGCAGCTCATC | + | 1^st^ cDNA reverse-transcried from *M. oryzae* Guy-  11 conidial total RNA | *MoVAC8* double stranded cDNA for complementation in yeast |
| Vac8yeast30.1 | *TCTAGA*TTAGCCTTCAATGTGTGATTTC | - | 1^st^ cDNA reverse-transcried from *M. oryzae* Guy-  11 conidial total RNA | *MoVAC8* double stranded cDNA for complementation in yeast |
| Tsc13yeast50.1 | *AAGCTT*ATGGCTGCAAGACTGAGCCTGAG | + | 1^st^ cDNA reverse-transcried from *M. oryzae* Guy-  11 conidial total RNA | *MoTSC13*  double stranded cDNA for complementation in yeast |
| Tsc13yeast30.1 | *TCTAGA*TTAAATGAGACCAGGAAGCATAG | - | 1^st^ cDNA reverse-transcried from *M. oryzae* Guy-  11 conidial total RNA | *MoTSC13*  double stranded cDNA for complementation in yeast |
| Vac8yEGFPFor | *GAATTC*ATGGGTGTTTGCAGCTCATCATG | + | 1^st^ cDNA reverse-transcried from *M. oryzae* Guy-  11 conidial total RNA | *MoVAC8* double stranded cDNA for *MoVAC8:yEGFP* |
| Vac8yEGFPRev | GTGAATAATTCTTCACCTTTAGACATGCCTTCAATGTGTGATTTCGAG | - | 1^st^ cDNA reverse-transcried from *M. oryzae* Guy-  11 conidial total RNA | *MoVAC8* cDNA for *MoVAC8:yEGFP* |
| Tsc13yEGFP-For | GAATTCATGGCTGCAAGACTGAGCCTG | + | 1^st^ cDNA reverse-transcried from *M. oryzae* Guy-  11 conidial total RNA | *MoTSC13* double stranded cDNA for *MoTSC13:yEGFP* construct |
| Tsc13yEGFP-Rev | GTGAATAATTCTTCACCTTTAGACATAATGAGACCAGGAAGCATAGTG | - | 1^st^ cDNA reverse-transcried from *M. oryzae* Guy-  11 conidial total RNA | *MoTSC13* double stranded cDNA for *MoTSC13:yEGFP* construct |
| yEGFP-For | ATGTCTAAAGGTGAAGAATTATTC | + | pKT127 [53] | yEGFP CDS for *MoVAC8:yEGFP* and *MoTSC13:yEGFP* constructs |
| yEGFP-Rev | *GCATGC*TTATTTGTACAATTCATCCATACC | - | pKT127 [53] | yEGFP CDS for *MoVAC8:yEGFP* and *MoTSC13:yEGFP* constructs |
| BAsplit | GGACTTCAGCAGGTGGGTGTAGAG | - | pCB1530 [49] | Spilt BA of selection marker gene *BAR* for targeted *MoATG4* gene deletion |
| ARsplit | GCAGACAGGAACGAGGACATTA | + | pCB1530 [49] | Spilt AR of selection marker gene *BAR* for targeted *MoATG4* gene deletion |
| Atg450.1 | tcaacaacgcagacagacacctca | + | *M. oryzae* Guy-  11 genomic  DNA | *MoATG4* RF for target gene deletion |
| Atg4m13f | gtcgtgactgggaaaaccctggcgacggcatttggggcaccttgtttg | - | *M. oryzae* Guy-  11 genomic  DNA | *MoATG4* RF for targeted gene deletion |
| Atg430.1 | gcaaagaaggacgacccgccaagt | - | *M. oryzae* Guy-  11 genomic  DNA | *MoATG4* LF for targeted gene deletion |
| Atg4m13r | tcctgtgtgaaattgttatccgcttcagggcaagtccatcattacagt | + | *M. oryzae* Guy-  11 genomic  DNA | *MoATG4* LF for targeted gene deletion |
